# Antimicrobial resistance reservoirs in salmon and broiler processing environments, sidestreams, and waste discharges

**DOI:** 10.3389/fmicb.2025.1662113

**Published:** 2025-09-16

**Authors:** Thorben Reiche, Anita Nordeng Jakobsen, Mihai Mares, Sunniva Hoel, Anne Tøndervik, Tonje Marita Bjerkan Heggeset, Tone Haugen, Andreas Husby Tømmerdal, Gunn Broli, Husnain Amir Butt, Iris Olene Bårdsen, Gunhild Hageskal

**Affiliations:** ^1^Department of Biotechnology and Food Science, Norwegian University of Science and Technology, Trondheim, Norway; ^2^Department of Public Health, “Ion Ionescu de la Brad” University of Life Sciences, Iași, Romania; ^3^Department of Biotechnology and Nanomedicine, SINTEF Industry, Trondheim, Norway

**Keywords:** chicken, poultry, aquaculture, rest raw material, wastewater, ARG, MDR, QIAseq xHYB

## Abstract

Mapping reservoirs of antimicrobial resistance (AMR) across food value chains and their environmental dissemination pathways is essential for limiting the spread and impact of AMR. The aim of this study was to investigate the prevalence of AMR genes and bacteria in sidestream materials, waste discharges, and processing environments of salmon and broiler. A targeted hybrid capture-based sequencing approach was used to characterize the resistome in samples collected from four processing plants, revealing a diverse range of AMR genes. Among these, we found several high-risk AMR genes, including the multidrug resistance genes *TolC* and *mdtE*, tetracycline genes *tet(L)* and *tet(M)*, aminoglycoside genes *APH(3′)-IIIa* and *APH(6)-Id*, and beta-lactam genes *mecA* and *mecR1*. Overall, the highest numbers of AMR genes were found in samples of process wastewater and sludge, ranging from 32 to 330 unique genes. More than 300 bacterial isolates, including Enterobacterales, *Enterococcus* and *Pseudomonas* spp. were also collected and identified, and a subset was tested for antibiotic susceptibility. Antibiotic resistance among *Enterococcus* and *Pseudomonas* spp. was low. Quinolone-resistant *Escherichia coli* (QREC) were detected in waste discharges from two broiler processing plants, while multidrug resistant (MDR) *E. coli* were found only in one plant. Whole genome sequencing of MDR isolates revealed multiple plasmids and AMR genes such as *sul2*, *ant(3″)-Ia*, *qnrS1*, and *bla_CTX-M-1_*. Our study highlights that wastewater from food industries can contribute to the release of AMR bacteria and genes to the environment. While the prevalence of AMR bacteria in sidestream materials was low among the isolates in our collection, numerous AMR genes were detected, which may be re-introduced to new production systems.

## 1 Introduction

Reducing the spread of antimicrobial resistance (AMR) is an ongoing challenge that requires a One-Health approach with coordinated actions from multiple sectors (ECDC and WHO, 2023). Food production systems are important linkages between humans, animals and the environment, with diverse pathways for potential AMR dissemination (Founou et al., 2016). As the AMR crisis continues to grow with a yearly estimated 1.27 million deaths globally (Murray et al., 2022), prevention strategies that limit spread and impact are important. Identifying AMR pathways and reservoirs can contribute to develop such strategies and to effectively implement new policies, biosecurity measures and hygiene procedures.

The broiler industry is among the largest food production systems in many European countries, including Romania, Poland and Hungary (Eurostat, 2020). Whereas the salmon industry is largest in Norway with an annual production of more than 1.5 million tons (Directory of Fisheries, 2022). On a global scale, the salmon industry produces nearly 3 million tons annually (Pandey et al., 2023), making it relatively small compared to the broiler industry, which produces around 120 million tons of meat each year (Oke et al., 2024). Both broiler and salmon are important sources of protein for the world’s growing population and make an increasing contribution to global food security (Neeteson-van Nieuwenhoven et al., 2013; Garlock et al., 2022). Ensuring sustainable production practices is crucial, as the supply chain for these industries continues to expand and become more sophisticated in line with globalization (Asche et al., 2018). This includes to minimize environmental impact, safeguard animal welfare, and use proactive disease management that controls the emergence of pathogens and AMR (Hafez and Attia, 2020).

Major transmission routes of AMR in salmon and broiler production systems include water, feed, humans, air dust and equipment (Koutsoumanis et al., 2021). Processing plants are key transmission routes between primary and secondary production. Inadequate processing hygiene and biosecurity can contribute to the release of AMR bacteria and genes to external environments or sectors through food products, sidestream materials, waste and waste discharges. Previous studies have indicated that process wastewater can be a hotspot for antibiotic resistant ESKAPEE bacteria (*Enterococcus faecium*, *Staphylococcus aureus*, *Klebsiella pneumoniae*, *Acinetobacter baumannii*, *Pseudomonas aeruginosa*, *Enterobacter* spp. and *Escherichia coli*) (Savin et al., 2020; Foyle et al., 2023). Among Norwegian broilers, an increase in quinolone resistant *E. coli* (QREC) occurrence has also been indicated (Kaspersen et al., 2018). Since the use of antibiotics for animals in Norway is low, non-antibiotic drivers such as biocides may play a greater role in AMR development. This includes disinfectants which may select for bacterial resistance, and studies have revealed correlations between antibiotic and disinfection resistance (Romero et al., 2017). In countries where antibiotic use is higher, such as Romania [European Centre for Disease Prevention and Control (ECDC), European Food Safety Authority (EFSA), European Medicines Agency (EMA), 2017], AMR bacteria are frequently detected (ANSVSA, 2022). A recent study by Brătfelan et al. (2023) found a high prevalence of tetracycline and ciprofloxacin (CIP) resistance among *E. coli* isolates from Romanian broiler meat. Whereas knowledge on AMR prevalence in the salmon industry is limited, a few studies have detected resistance among resident *Pseudomonas* and *Aeromonas* spp. (Lee et al., 2021; Thomassen et al., 2022).

Antimicrobial resistance genes (ARGs) play a central role in the spread of AMR across bacterial populations through horizontal gene transfer (HGT) (Fletcher, 2015). ARGs have been detected in a wide range of food industry compartments including food products (Doster et al., 2020), air dust (Luiken et al., 2022), waste (Lin et al., 2022) and processing environments (Cobo-Díaz et al., 2021; Merino et al., 2024). Genes that pose a threat to human health include those that confer resistance to clinically relevant antibiotics, and have strong host pathogenicity and mobility with high likelihood of transmission between bacteria (Zhang et al., 2021, 2022). For example, *tet(M)* is considered as a high-risk ARG because it confers resistance towards clinically relevant tetracycline, has high mobility rates and is present among ESKAPEE bacteria. While the occurrence of AMR bacteria in humans and animals is actively monitored by national agencies in Norway and Romania (ANSVSA, 2022; NORM/NORM-VET, 2023), knowledge on ARG prevalence in environmental DNA samples from food, processing plants and wastes is limited. There is also a lack of knowledge on sidestream materials as potential transmission routes for AMR. Increased focus on circular economy and resource exploitation is promoting the production of feed ingredients using residual raw materials from broiler and salmon.

Here, we characterize resistomes in sidestream materials, waste discharges and processing environments of salmon and broiler using a targeted hybrid capture-based sequencing approach. For this analysis, we collected samples from three processing plants in Norway and one in Romania. A large collection of bacterial isolates including Enterobacterales, *Enterococcus* and *Pseudomonas* spp. was also tested for antibiotic susceptibility. Furthermore, we analyze *E. coli* genotypes with high phenotypic resistance profiles and determine the prevalence of ESBL producing isolates and QREC-isolates among these. Finally, we investigate disinfectant adaptation in *Pseudomonas*-isolates after prolonged exposure towards sub-inhibitory concentrations.

## 2 Materials and methods

### 2.1 Sampling locations and collection techniques

This study investigates processing plants of salmon (Plant A), sidestream materials (Plant B), and broiler (Plant C) located in Norway and one broiler processing plant in Romania (Plant D). The production is highly automated in these plants with annual processing capacities of approximately 150,000 tons of salmon (Plant A), 40,000 tons of sidestream materials (Plant B), and more than 100,000 broilers per day (Plant C and D). The sidestream materials processed in Plant B are primarily derived from salmon production in Plant A. The processing of broiler sidestream materials is currently in the testing-phase.

Processing Plants A, B, C and D have in-house treatment systems for wastewater from the processing environment. Large particles are separated by filters in the production environment, and sludge is removed from wastewater in large flotation units. Additional water treatment differs between plants. In Plant A, seawater is used in electrolysis cells to produce chlorine, which is then added to wastewater and passed through holding loops. The pH level in the wastewater is also adjusted. In Plant B, wastewater is disinfected by the addition of hypochlorite, while Plant C adjusts wastewater pH with lye (hydroxide). After the treatment, Plant A, B, and C release the wastewater to the nearby fjord. Plant D, on the other hand, releases the wastewater to the municipal sewage system for further treatment.

**Figure 1:**
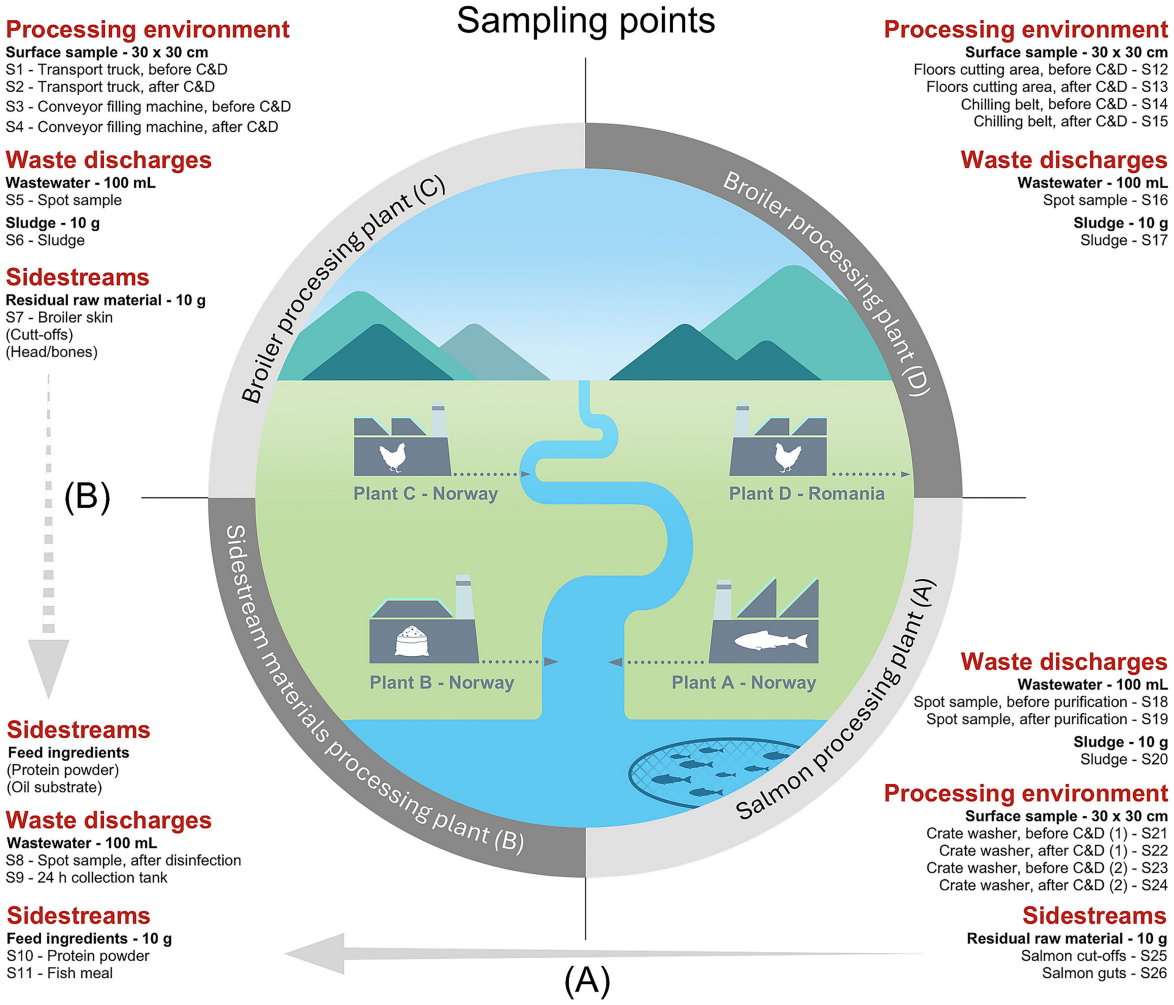
Overview of sampling points in processing plants of salmon (Plant A), sidestream materials (Plant B) and broiler (Plant C and Plant D) in sample categories: processing environment, waste discharges, and sidestreams. The processing environment includes areas both involved in pre-processing (e.g., transport truck floors for live animals) and processing (e.g., surfaces in cutting, washing, and packaging areas). Plants A, B and C (Norway) release their wastewater to the nearby fjord after purification, while Plant D (Romania) discharges wastewater to the municipal sewage system. Samples from the process environment were taken before and after cleaning and disinfection (C&D). The crate washer in Plant A was sampled both during the cold season (1) and warm season (2). **(A)** Salmon residual raw materials from Plant A are transferred to Plant B for feed ingredient production. **(B)** Indicates a future potential to produce feed ingredients from broiler residual raw materials.

Samples of wastewater (100 mL) and sludge (10 g) were collected using sterile containers. In Plant A, wastewater was sampled both before and after purification in the chlorine holding loops, using five replicates. In Plant B, ten replicates of wastewater were collected before disinfection by hypochlorite from a wastewater collection tank, which collects small amounts of wastewater over a 24 h period. Ten additional replicates of wastewater were collected by spot sampling after disinfection. Ten replicates of wastewater were also collected in Plant C and D. Sludge samples were collected with ten replicates in all processing plants except Plant B. Sidestream materials (10 g) were provided by Plant A, B, and C with ten replicates and included residual raw materials of salmon such as cut-offs and guts (Plant A), broiler skin (Plant C), and pet feed ingredients made from salmon including fish meal and protein powder (Plant B). Cut-offs generally refer to various parts of the salmon fillet that are removed during the trimming process, such as the belly fat, collarbone, backfin, and tailpiece. All samples were kept cold during transportation to the laboratory. Initial sample processing, plating on selective media and total DNA extraction from Plant D samples were carried out at the laboratory of IULS. All remaining analyses were performed at laboratories of SINTEF or NTNU. Sludge and sidestream samples (10 g) were diluted with 90 mL of peptone water (1 g/L bacteriological peptone, 8.5 g/L NaCl) and homogenized in a Stomacher 400 Lab Blender (Seward Medical Ltd., London, UK) for 1 min. Additional samples (eDNA) were obtained from our previous studies by Reiche et al. (2024, 2025). These included cloth surface samples taken in the production environment of Plant A, C, and D before and after cleaning and disinfection (C&D). Complete overview of sampling points is given in Figure 1.

### 2.2 Detection of antibiotic resistance genes (ARGs)

For DNA extraction from wastewater, falcon tubes with 2 × 50 mL were centrifugated, i.e., 50 mL were centrifuged, and the supernatant was removed, then new 50 mL were added to the same tube and centrifuged again. The centrifugation conditions were 2,700 g for 10 min. Homogenates of sludge and sidestream materials were centrifugated in lower volumes of 10 mL. The supernatant was removed, and total DNA was isolated using the remaining pellets from one replicate of each sample type. Samples from Plant A, B and C were subjected to the MasterPure Gram Positive DNA Purification Kit (Biosearch Technologies, Novato, USA), while samples from Plant D were subjected to the OmniPrep kit for Gram-positive bacteria (G-Biosciences, St. Louis, USA). DNA extraction was performed according to the manufacturer’s protocols. DNA quantification and quality assessment was performed on a Qubit 2.0 fluorometer using the Qubit dsDNA BR Assay Kit (Thermo Fisher Scientific, Eugene, USA) and a NanoDrop UV–Vis Spectrophotometer (Thermo Fisher Scientific, Wilmington, USA).

Sequencing libraries were generated from eDNA using the QIAseq FX DNA Library Kit (Qiagen, Hilden, Germany) where unique dual index (UDI) adapters were included and used for multiplexing. Libraries were pooled and enriched for ARGs by multiplex hybrid capture using the QIAseq xHYB AMR Panel Kit (Qiagen, Hilden, Germany). In brief: Purified libraries were pooled and an enhanced blocking buffer added to prevent non-specific hybridization. Libraries were concentrated using QIAseq beads, mixed with the QIAseq xHYB probe panel and incubated at 70 °C for 16 h. Hybridized libraries were captured on streptavidin beads, and washed twice to remove unbound DNA. After elution, the enriched libraries were PCR amplified for 20 cycles. The final library pool was sequenced on an Illumina MiSeq using the MiSeq Reagent kit v2 in the 2 × 150 bp PE mode (Illumina Inc., San Diego, USA).

Data was basecalled, demultiplexed and converted to fastq in MiSeq Reporter (Illumina). Data was then analyzed using the “Find QIAseq xHYB AMR Markers” workflow in CLC Genomics Workbench v. 24.0 (Qiagen) using default parameters. The Qiagen QMI-AR Peptide Marker Database (v. 2021–08) (qmi_ar_peptide_marker_database_2021_08), which covers 3,622 antimicrobial resistance genes was used as recommended. The database contains peptide markers derived from the following sources: CARD, ARG-ANNOT, NCBI, ResFinder. In brief: raw reads were trimmed, removing adapter sequences and low-quality nucleotide sequences. Resistance genes were then detected and quantified using the ShortBRED algorithm (Kaminski et al., 2015) with the genetic code set to 11 (Bacterial, Archaeal and Plant Plastid), E-value at 0.00001, 95% identity, minimum alignment length 0.95, minimum read length 90.0, and running in the more sensitive search mode. Data were reported in number of reads as well as normalized abundance in RPKM (Reads Per Kilobase per Million reads). Plots and heatmaps were generated by R package ggplot2 (v. 3.5.1) (Wickham, 2011).

### 2.3 Isolation and identification of bacterial isolates

Bacterial isolates were collected using selective media targeting quinolone resistant bacteria and extended spectrum beta-lactamase (ESBL)-producing bacteria. Replicates of homogenates from wastewater, sludge and sidestreams from Plant A, B, and C were plated on MacConkey Agar (MAC; Oxoid, Basingstoke, UK) with 1 mg/L cefotaxime (CTX) and 0.06 mg/L CIP (Sigma-Aldrich, Schnelldorf, Germany), respectively. To increase the detection limit, a 1 mL aliquot was evenly distributed across three agar plates. Incubation conditions were 41 ± 0.5 °C for 18–22 h. Colonies were then re-streaked on MAC with 2 mg/L CTX and 0.5 mg/L CIP, while homogenates from Plant D were plated directly on these concentrations and incubated at the same conditions. Up to 4 colonies from each replicate were re-streaked minimum twice on Tryptic Soy Agar (TSA; VWR, Leuven, Belgium) and incubated at the same conditions. Glycerol stocks were prepared using Tryptic Soy Broth (TSB; VWR, Leuven, Belgium) with 20% glycerol and stored at −80 °C.

DNA was isolated from 324 bacterial isolates (Supplementary Table S1) using the Genomic Micro AX Bacteria Gravity Kit (A&A Biotechnology, Gdynia, Poland) following the manufactures protocol. The polymerase chain reaction (PCR) was performed for the 16S rRNA gene (V3-V9) using universal primers, with PCR conditions available in Supplementary Table S2. Amplicons were purified using ExoSap-IT (Thermo Fischer Scientific, Waltham, USA) and Sanger sequenced by Eurofins Genomics (Köln, Germany). *In-silico* sequences were analyzed in the GenBank database using BLASTn by the National Center for Biotechnology Information (NCBI).

### 2.4 Antibiotic susceptibility testing

Robotic high-throughput screening (HTS) was performed to test antibiotic susceptibility in two selected groups of bacterial isolates: (1) Enterobacterales, *Aeromonas* and *Kurthia* isolates (*n* = 45), and (2) *Pseudomonas* and *Acinetobacter* isolates (*n* = 32). Each group was tested towards the same antibiotics (*n* = 9) using lower concentrations for the former (1) and higher concentrations for the latter (2) (concentrations shown in Section 3.4 Antibiotic susceptibility in isolates from waste discharges and sidestream materials).

An Echo 650 series acoustic liquid handler (Beckman Coulter Inc., Brea, USA) was used to prefill sterile Nunc flat-bottom 384-well plates (Thermo Scientific, New York, USA) with antibiotic stock solutions. In brief, 4.5 mL tubes were used to prepare initial stock solutions before they were aliquoted to 1.8 mL and transferred to the Echo liquid handler, which dispenses droplets of 2.5 nanoliters to well plates. These volumes were adjusted according to the desired final concentration in the assay. Prefilled plates were sealed and kept cold before inoculation. The HTS protocol was carried out by a Biomek i7 Automated Workstation (Beckman Coulter Inc., Brea, USA) performing pipetting-steps with high accuracy, facilitating reproducibility and standardization. *E. coli* reference strains ATCC 25922, CCUG 59342 (ESBL positive) and CCUG 59351 (AmpC positive) were included in the assay, in addition to *P. aeruginosa* ATCC 27853.

Precultures were prepared by adding 50 μL of bacterial glycerol stock to 450 μL of Tryptone Soya Broth (TSB; Oxoid, Basingstoke, UK) in 96-deep well plates (Masterblock, 2 mL, V-bottom) (Greiner Bio-one, Stonehouse, UK). Incubation conditions were 37 °C at 85% humidity and 900 rpm (25 mm amplitude) for 18–22 h. Overnight cultures were mixed by aspirating and dispensing 150 μL ten times both before and after an 800-fold dilution was performed using Mueller Hinton II broth cation adjusted (MH; Becton, Dickinson and Company, New Jersey, USA) in 96 deep well plates. Prefilled 384-plates with antibiotic stock solutions were inoculated in triplicates with 37.5 μL of the diluted cultures and shaken at 2000 rpm for 20 s. Ziplock bags were used to seal the plates during statical incubation at 37 °C for 24 h. Plates were shaken at 2000 rpm for 20 s prior to optical density (OD_600_) measurements in a Tecan Spark microplate reader (Tecan, Männedorf, Switzerland). Minimum inhibitory concentrations (MICs) were read as the lowest concentration that inhibits ≥70% of growth (OD_600_) compared to the growth control. Plots were generated by R package ggplot2 (v. 3.5.1) and ggpubr (v. 0.6.0) (Kassambara, 2025).

Additional tests were performed with *E. coli* isolates (*n* = 22) from group (1) to investigate their ESBL-phenotype and to confirm their CIP susceptibility using a broader range of concentrations than previously tested. Reference strains used in the HTS assay were also included in these tests. For the ESBL detection, Sensititre EU Surveillance ESBL EUVSEC2 AST plates (Trek Diagnostic Ltd., East Grinstead, UK) were used and inoculated according to the manufacturer’s protocol. The CIP assay was manually prepared following a procedure similar to the Sensititre protocol. In brief, Nunc flat-bottom 96-well plates (Thermo Scientific, Roskilde, Denmark) were prefilled with 100 μL cation-adjusted MH broth (Oxoid, Basingstoke, UK) containing two-fold concentrations of CIP. Inocula were prepared by transferring a few colonies from overnight cultures on TSA to sterile demineralized water and by standardizing to 0.5 MacFarland. Next, 60 μL of the standardized suspensions were diluted in 11 mL cation-adjusted MH broth. CIP plates were inoculated by 100 μL of cultures in triplicates with final CIP concentrations of 0.5–16 mg/L. Plates were sealed and incubated at 37 °C for 18–24 h.

*Enterococcus* isolates (*n* = 67) were tested using Sensititre EU Surveillance Enterococcus EUVENC AST plates (Sensititre, Trek Diagnostic Systems Ltd., East Grinstead, UK). The method was performed according to the manufacturer’s protocol with twelve antimicrobials in different concentrations (concentrations shown in Section 3.4 Antibiotic susceptibility in isolates from waste discharges and sidestream materials). Reference strain *E. faecalis* ATCC 29212 was included in the assay. MICs for EUVSEC2, CIP and EUVENC were read manually using the Thermo Scientific Sensititre Manual Viewer with a mirrored viewbox. Resistance was defined based on EUCAST epidemiological cut-off values (ECOFFs) (Supplementary Table S3). Multidrug resistance was defined as non-susceptible to ≥1 agent in >3 antimicrobial categories (Magiorakos et al., 2012).

### 2.5 Whole genome sequencing of *Escherichia coli* isolates

A selection of ten *E. coli* isolates with high levels of phenotypic resistance were subjected to whole genome sequencing. Previously isolated DNA (Section 2.3 *“Isolation and identification of bacterial solates”*) was quantified and quality assessed on a Qubit 2.0 fluorometer. Sequencing libraries were generated using the Nextera XT DNA library preparation kit (Illumina Inc., San Diego, USA) in combination with an Illumina dual index kit for multiplexing. Libraries were pooled and sequenced on an Illumina MiSeq sequencer using the MiSeq Reagent kit v3 in the 2 × 300 bp PE mode.

Data was base-called, demultiplexed and converted to fastq in MiSeq Reporter (Illumina), and draft assemblies generated in CLC Genomics workbench v. 25.0 (Qiagen). In brief: Raw sequencing reads were quality assessed using the Trim Reads tool (v. 3.0), removing adapter sequences and sequences with more than 2 ambiguous bases. Trimmed reads were then assembled using the *De Novo* Assembly (v. 1.5) tool in the “map reads back to contigs” mode. Due to the overall low sequencing depth (22.6–38.8 × on average) the assemblies were filtered by removing contigs shorter than 200 bp and those resulting from less than 10 individual reads. Finally, the NCBI Foreign Contamination Screen tool (Astashyn et al., 2024) was run prior to submission to NCBI, removing any contigs from contaminating cells or viruses. Assembled genomes were analyzed by services provided from the Center for Genomic Epidemiology using standard settings: SerotypeFinder 2.0.1 (Joensen et al., 2015), MLST 2.0.9 (Wirth et al., 2006; Camacho et al., 2009; Larsen et al., 2012), cgMLSTFinder 1.0.1 (Clausen et al., 2018; Zhou et al., 2020), VirulenceFinder 2.0.5 (Camacho et al., 2009; Joensen et al., 2014; Malberg Tetzschner et al., 2020), PathogenFinder 1.1 (Cosentino et al., 2013), PlasmidFinder, 2.0.1 (Camacho et al., 2009; Carattoli et al., 2014), MGE 1.0.3 (Johansson et al., 2020) and ResFinder 4.7.2 (Camacho et al., 2009; Bortolaia et al., 2020).

### 2.6 Development of bacterial tolerance towards disinfectants

A selection of isolates (*n* = 16) was cultivated in the presence of increasing concentrations of a commercial disinfectant using a BioLector I microbioreactor (Beckman Coulter Inc., Brea, USA). The instrument measures light scattering in well plates to quantify the isolates’ cell biomass during disinfectant-exposure. The selection of bacteria (Supplementary Table S4) included 14 isolates classified in the *Pseudomonas fluorescens* group, one *Aeromonas*-isolate and one *Serratia*-isolate, which were obtained from our previous study by Reiche et al. (2024, 2025) and originated from processing Plants A, C and D.

Glycerol stocks were partly thawed, and approximately 20 μL were transferred to 50 mL falcon tubes containing 6 mL of Tryptone Soya Broth (TSB; Oxoid, Basingstoke, UK). Incubation was performed overnight at 25 °C and 200 rpm shaking (orbital diameter 2.54 cm). The commercial disinfectant Aqua Des Foam PAA (Aquatic Chemistry AS, Lillehammer, Norway) based on peracetic acid and hydrogen peroxide was included in the experiment. The disinfectant was diluted in Mueller Hinton II broth cation adjusted (MHB; Becton, Dickinson and Company, New Jersey, USA) equivalent to 1/8 of the isolates’ MIC values previously determined by Reiche et al. (2024); (2025). The experiment was performed using either 48- or 96-well plates (BioLector 48 Round Well, Beckman Coulter Inc., Brea, USA; VWR 96 Round Well, PS, Flat bottom, Black, VWR, Leuven, Belgium). The plates were prefilled with 900 and 185 μL of MHB-disinfectant solution and inoculated by cultures in triplicates using 30 and 15 μL for 48- and 96-well plates, respectively, giving a start-OD_600_ of 0.1 in the well plates. Incubation was performed with shaking at 1200 rpm (48-well plates) or 700 rpm (96-well plates) at 25 °C for 24–48 h depending on the growth, i.e., replicates with little or no growth were incubated longer. After incubation, cultures with growth were preserved as glycerol stocks and used to inoculate fresh MHB-disinfectant solution in well plates. Disinfectant concentrations were gradually increased for each cycle of re-inoculation and incubation until complete inhibition was achieved.

Populations with growth in disinfectant concentrations above MIC were subjected to a verification test comparing the original wild-type strain to the disinfectant-adapted strain. Overnight TSA plates (25 °C) were prepared from glycerol stocks. Four random colonies from each plate were transferred to individual falcon tubes containing 6 mL of TSB and incubated using the same conditions as before. Cultures from single colonies were inoculated in triplicates to 96-well plates prefilled with a gradient of MHB-disinfectant solution, giving a start-OD_600_ of 0.1. Incubation was performed with the same conditions as before. The BioLector software (BioLection v. 2.6.0.0) was used to export Microsoft Excel datasheets from the BioLector. A gain value (G) of 35 was applied, which the software uses to amplify the detected light signal and estimate cell biomass. Plots were generated by R package ggplot2 (v. 3.5.1). A subset of isolates with verified disinfectant-adaptation was subjected to robotic HTS antibiotic susceptibility testing using the same materials and method as before (Section 2.4 *“Antibiotic susceptibility testing”*) with incubation at 25 °C. Both the wild-type and disinfectant-adapted strains were included.

## 3 Results and discussion

### 3.1 Resistomes in waste discharges and sidestream materials from broiler and salmon

**Figure 2:**
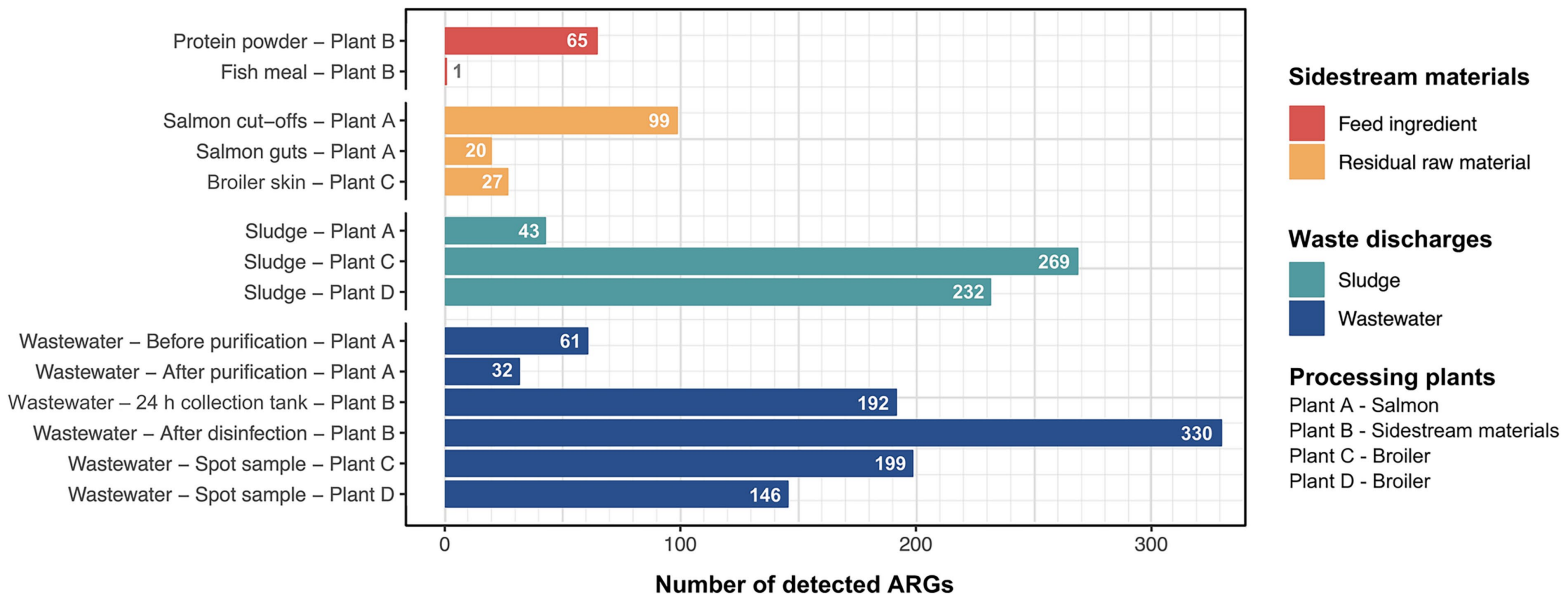
Number of unique antimicrobial resistance genes (ARGs) detected in sample categories of sidestream materials and waste discharges from processing plants of salmon (Plant A), sidestream materials (Plant B) and broiler (Plant C and Plant D).

To assess the risk of AMR dissemination by waste discharges and sidestream materials from broiler and salmon industries, we performed a targeted hybrid capture-based sequencing method. All samples had reads that aligned to at least one ARG and the average number of aligned reads was approximately 5 × 10^4^. A total of 434 unique ARGs were detected across all sample types, ranging from 1 to 330 ARGs per sample (complete list in Supplementary Table S5). The number of ARGs was considerably lower in sidestream materials (1–99) compared to waste discharges (32–330) as we expected (Figure 2).

#### 3.1.1 Residual raw materials and feed ingredients

**Figure 3:**
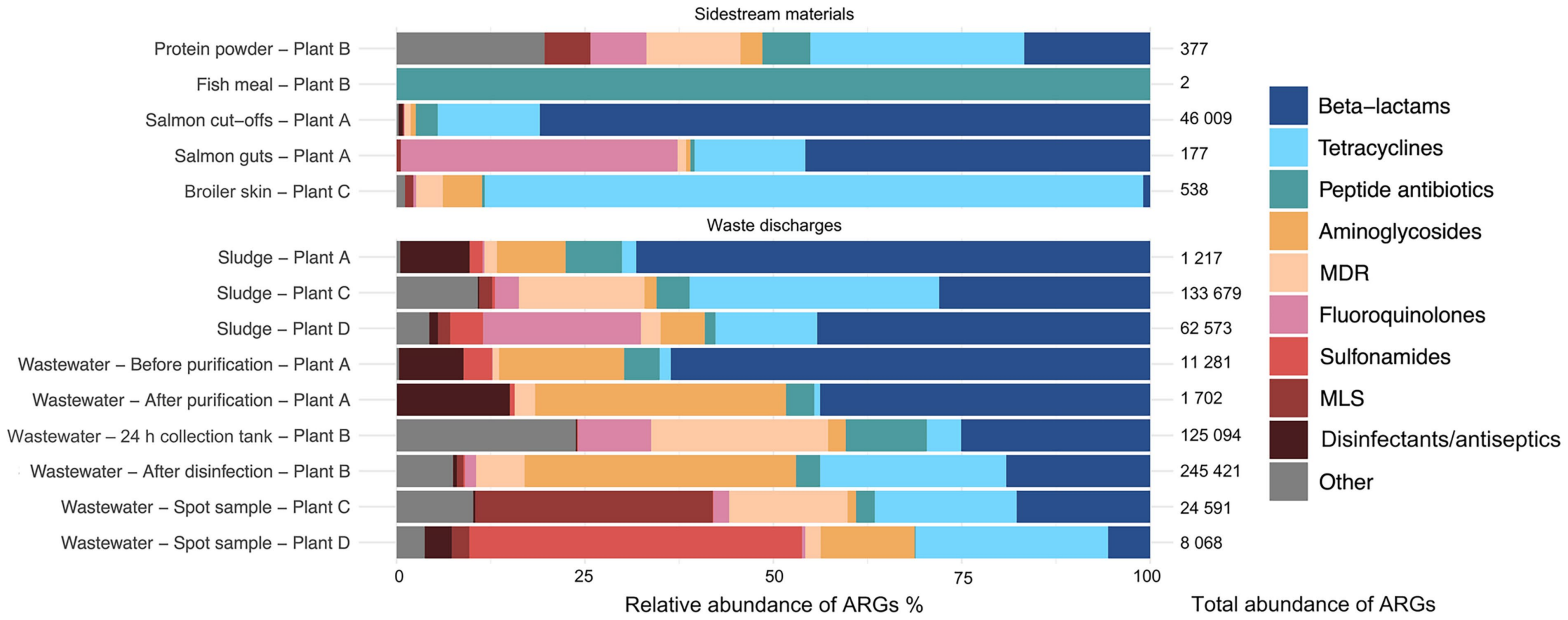
Relative abundance (%) of antimicrobial resistance genes (ARGs) assigned to di!erent antimicrobial classes in sample categories sidestream materials and waste discharges from processing plants of salmon (Plant A), sidestream materials (Plant B) and broiler (Plant C and Plant D). Abbreviations MLS for Macrolide, Lincosamide, Streptogramin, and MDR for multidrug resistance.

**Figure 4:**
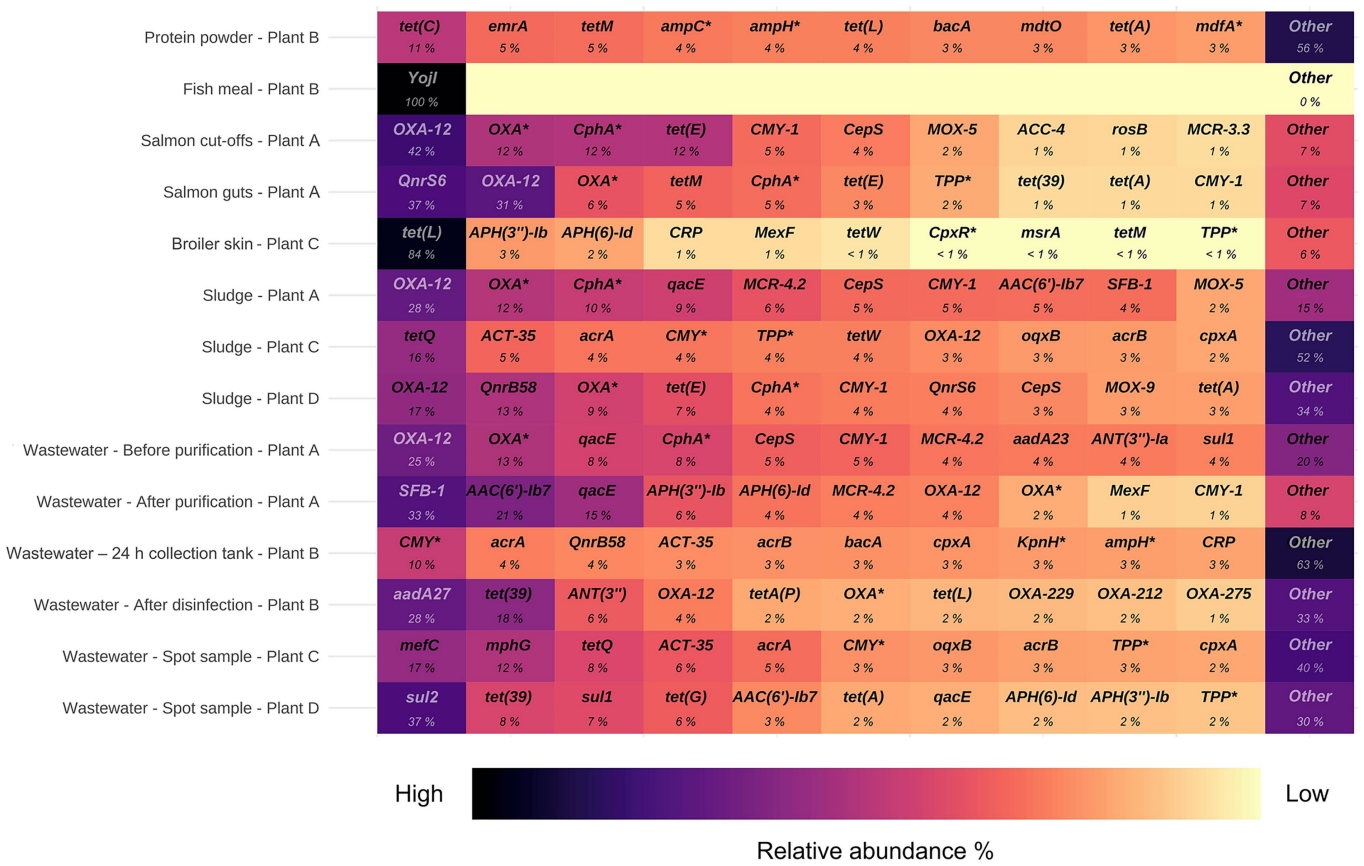
Top ten antimicrobial resistance genes (ARGs) with highest relative abundance in samples from processing plants of salmon (Plant A), sidestream materials (Plant B) and broiler (Plant C and Plant D). Scalebar indicates relative abundance % from high (black) to low (yellow). ARGs marked with an asterisk are abbreviated: OXA* (*OXA* beta-lactamase), CphA* (*CphA* beta-lactamase), ampC* (*E. coli ampC* beta-lactamase), ampH* (*E. coli ampH* beta-lactamase), mdfA* (*E. coli mdfA*), CMY* (*CMY* beta-lactamase), KpnH* (*K. pneumoniae KpnH*), TPP* (tetracycline-resistant ribosomal protection protein), CpxR* (*P. aeruginosa CpxR*).

Among the residual raw materials, salmon cut-offs had the highest number of detected ARGs (*n* = 99), which corresponded primarily to the beta-lactam resistance class with a relative abundance of 81% (Figure 3), followed by tetracycline (14%) and peptide antibiotic (3%) resistance classes. The cloxacillin-hydrolyzing enzyme-encoding gene *OXA-12* showed the highest relative abundance (42%) (Figure 4). This gene has previously been described in *Aeromonas* spp. isolated from Norwegian retail sushi (Lee et al., 2023). Interestingly, the study also found *tet(E)*, *cepS* and *cphA*-genes in the *Aeromonas* isolates, all of which were among the top ten most abundant ARGs in the salmon cut-offs. The *cphA*-genes have previously been reported as intrinsic in environmental *Aeromonas* spp. and encode carbapenem-hydrolyzing enzymes (Balsalobre et al., 2009). In the salmon guts, *OXA-12, tet(E)* and *cphA* were also among the top ten ARGs with *qnrS6* having the highest relative abundance (37%). *qnrS6* confers resistance towards quinolones of which oxolinic acid is the second most used antibiotic in the Norwegian salmon aquaculture, although it is only used in small quantities (NORM/NORM-VET, 2023). Previous studies have found a variety of *qnr*-genes in bacterial strains isolated from Chilean aquaculture farms. As the historical use of oxolinic acid in Chilean aquaculture has been high, the authors corroborated that the usage may have evolved quinolone resistant bacteria (Buschmann et al., 2012; Valdes et al., 2015). However, more recent data shows that farmers have stopped using oxolinic acid in Chile (Miranda et al., 2018). Moreover, we observed that the total number of ARGs was considerably lower in the salmon guts (20) compared to the salmon cut-offs (99) indicating that the salmon gut resistome is less diverse. The cut-offs typically consist of fat and muscle tissue, in addition to smaller parts of skin or bones removed during filet trimming. Since the salmon flesh is sterile or nearly sterile, the high number of ARGs may originate from skin microbiota or processing related cross-contamination. Overall, our results show that salmon residual raw materials harbor a variety of ARGs which may be re-introduced into new production systems.

In the residual raw material of broiler skin, we detected 27 ARGs, of which tetracycline resistance genes dominated (87%), with *tet(L)* being most abundant. ARGs in the class of aminoglycosides (5%), including *APH(3″)-Ib* and *APH(6)-Id* were also found, in addition to multidrug resistance (MDR) genes (4%). Similar results have been reported for chicken thighs sampled from a poultry processing line using a different sampling and sequencing method (Merino et al., 2024). The study found between 10–20 ARGs per sample with genes in resistance classes of tetracyclines and aminoglycosides in high abundances. Prior to slaughter, the skin of broilers is a bacterial hotspot and often contaminated by fecal content (Marmion et al., 2021). However, the microbiome is altered considerably during scalding, where whole broilers are submerged in a water bath for 133 s at 55 °C (Plant C), reducing bacterial loads and re-shaping the microbiota (Hauge et al., 2023). Effective scalding, combined with post-scalding practices that minimize cross-contamination, likely contributes to controlling the resistome. This may explain the lower number of detected ARGs in the broiler skin compared to the salmon cut-offs. Maintaining low ARG levels is important for enabling the safe use of residual raw materials in the production of feed ingredients.

Our results indicate that feed ingredients may serve as reservoir for ARGs. Although only one gene was detected in the fish meal, 65 ARGs were found in the salmon protein powder. The efflux pump encoding gene *YojI* conferring resistance to peptide antibiotics was present in the fish meal. While in the protein powder, we detected genes associated with classes of tetracycline (28%), beta-lactams (17%) and MDR (13%). ARGs with highest relative abundance were *tet(C), emrA, tet(M), ampC* and *ampH*. The production of both fish meal and protein powder involves extensive processing including grinding, enzymatic hydrolysis, heating and drying, keeping bacterial survival to a minimum. Genetic elements including ARGs can tolerate higher temperatures and may endure the harsh processing conditions (James et al., 2021). The difference in ARG prevalence between protein powder and fish meal may be explained by the drying conditions. Protein powder is spray dried with temperatures above 100 °C for a few seconds, while fish meal is disk dried at lower temperatures for 4–6 h. Nevertheless, post-processing cross-contamination cannot be ruled out completely. A previous study investigated commercial fish meal made by cod and anchovies, and found 3–14 unique ARGs per sample (Han et al., 2019). Comparable studies investigating ARGs in salmon sidestream materials do not exist to the best of our knowledge. In this study, the sample size is relatively small. Further investigations are therefore needed to assess the risk of AMR dissemination more accurately in both residual raw material and feed ingredients.

#### 3.1.2 Sludge and process wastewater

Among the waste discharges, we observed large differences in the number of detected ARGs between sludge from salmon and broiler processing plants (Figure 2). While 269 and 232 ARGs were found in sludge from broiler processing Plants C and D, respectively, only 32 genes were detected in sludge from salmon processing Plant A. Similar ARGs were found in the sludge from the salmon processing plant as in the sidestream materials of salmon. These include *OXA-12, CphA,* and *CepS,* making beta-lactams the dominant resistance class (68%) (Figures 3, 4). A higher relative abundance of genes associated with disinfectant resistance was also found in the sludge compared to the sidestream materials, primarily *qacE.* Chemical residues from cleaning and disinfection can impact the resistome (Xiao et al., 2024) and quaternary ammonium compound (QAC) resistance genes are commonly found in foodborne pathogens (Daeschel et al., 2022). Nevertheless, QACs are not used as disinfectants in the Norwegian food industry anymore. In sludge from the broiler processing plants, we detected a higher percentage of ARGs in classes of tetracycline including *tet(Q), tet(E)* and *tet(A),* and several MDR genes in sludge from Plant C. These findings highlight the need for waste treatment operators to consider the potential risk of AMR dissemination from sludge wastes.

The process wastewater from the broiler processing Plants C and D had lower numbers of ARGs (199 and 146) compared to their respective sludge sample (>230). This was expected since ARGs tend to accumulate in the sludge, leading to lower levels of genes in wastewater (Wang et al., 2022). ARGs in classes of MLS (macrolide, lincosamide, streptogramin) (32%), tetracyclines (19%) and beta-lactams (18%) dominated in wastewater from Plant C, with *mefC, mphG* and *tet(Q)* being most abundant. A high abundance of sulfonamide genes (44%), such as *sul1* and *sul2,* was found in wastewater from Plant D, followed by aminoglycoside (26%) and tetracycline (13%) genes. Sales data on the antibiotics used in Romania and Norway for food-producing animals show substantial differences (EMA, 2022). While 49 mg of antibiotics per PCU (population correction unit) were sold in Romania, only 2 mg/PCU were sold in Norway. Among waste discharges from the broiler processing plants, we found ARGs in classes of the most sold antibiotics in both Norway and Romania. These include beta-lactams and tetracyclines in both countries, in addition to fluoroquinolones that are widely used in Romania. A previous study investigating Romanian poultry meat reported that the significant use of fluoroquinolones has led to the emergence of quinolone-resistant *Campylobacter jejuni*, *E. coli* and *Salmonella enterica* serovar Enteritidis (Dan et al., 2015). Furthermore, wastewater from the salmon processing Plant A had the lowest number of ARGs (61), with a 50% reduction in the number of genes after purification. The highest number of ARGs (330) was detected in wastewater sampled after disinfection by hypochlorite from the sidestream material processing Plant B. ARGs in the class of aminoglycosides showed the highest relative abundance (44%) including *aadA27* and *ANT(3″).* The number of ARGs was lower (192) in the wastewater from the 24 h collection tank. This was unexpected since the collection tank was sampled prior to disinfection. The long retention time in the collection tank may allow microbial degradation of some ARGs. Additionally, the spot sample after disinfection represents a single moment in time, potentially capturing a high ARG peak, while the collection tank sample represents an average over time, which can smooth out fluctuations and result in an overall lower ARG count. Overall, our findings show that process wastewater from food processing plants contributes to the spread of AMR genes. The release of wastewater to surrounding fjords by the Norwegian processing plants is concerning, and wastewater treatment should prevent bacteriological pollution, including harmful bacteria and genes.

#### 3.1.3 High-risk antimicrobial resistance genes (ARGs)

**Figure 5:**
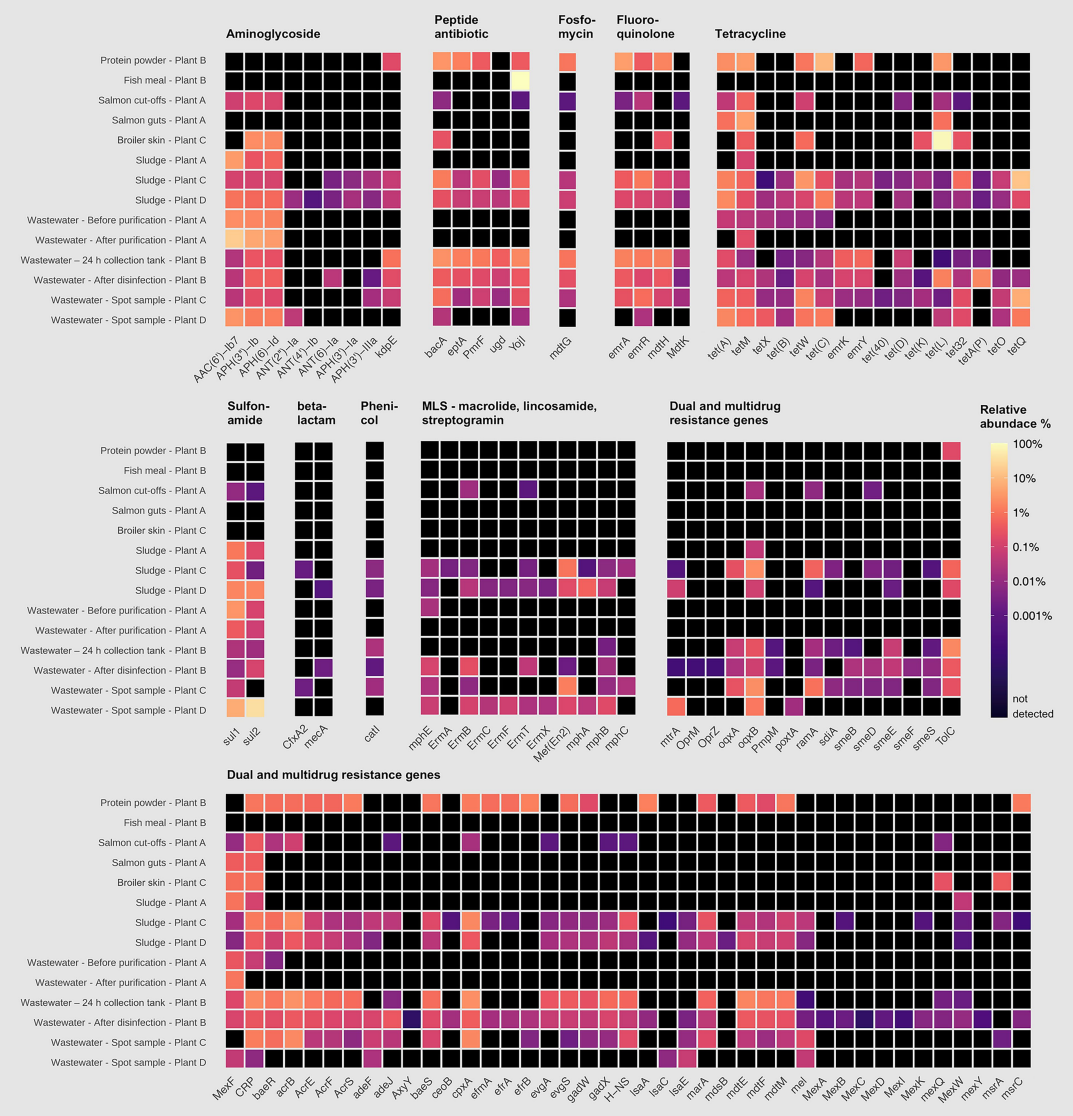
Relative abundance (%) of high-risk antimicrobial resistance genes (ARGs) in samples from processing plants of salmon (Plant A), sidestream materials (Plant B) and broiler (Plant C and Plant D). Scalebar indicates relative abundance % from high (yellow) to low (purple), where black indicates that the ARG was not detected.

To evaluate the health risk of the detected ARGs, we applied the criteria proposed by Zhang et al. (2022) which classify genes by resistance to clinically relevant antibiotics, host pathogenicity, human accessibility and mobility with high likelihood of transmission between bacteria. The authors classified more than 2,500 genes into two main groups: ARGs with health risk (1) and ARGs with no health risk (2). The first group was divided into sub-categories Q1, Q2, Q3 and Q4 ranging from low to high health-risk. A total of 152 ARGs were placed in the high-risk group (Q1), all of which are found among pathogenic bacteria. In this study, we found 107 out of these 152 high-risk genes across the sample types of sidestream materials and waste discharges (Figure 5). Overall, more than 50% of the detected high-risk genes were dual and multidrug resistance genes. Sludge from broiler processing Plants C and D, and wastewater from the sidestream material processing Plant B were among the samples with the largest number of high-risk genes (≥60). For example, we detected genes encoding multidrug efflux pumps, such as *TolC*, *OqxB* and *adeJ,* in these samples. *TolC* is a subunit of many multidrug efflux complexes, such as *AcrAB-TolC,* and has been reported to confer resistance to nearly all common antibiotics (macrolide, fluoroquinolone, aminoglycosides, carbapenem, cephalosporin, glycylcycline, cephamycin, penam, tetracycline, peptide antibiotic, aminocoumarin, rifamycin, phenicol, triclosan, penem) (Zhang et al., 2022). Likewise, *OqxB* confers resistance to many important antibiotics, is present in pathogens such as *E. coli, K. pneumoniae* and *Enterobacter cloacae*, and is also present on mobile insertion elements or plasmids (Bharatham et al., 2021). The *adeJ* gene is a part of the *AdeIJK* efflux complex in *A. baumannii* and a 4-fold to 16-fold MIC decrease with levofloxacin, chloramphenicol, doxycycline, tetracycline and tigecycline has been reported upon gene-knockout (Xie et al., 2025). *mecA*, responsible for methicillin resistance in *S. aureus* (MRSA) was also detected in sludge from Plant D and wastewater from Plant B. Ultimately, we found a wide variety of high-risk ARGs and the spread of these should be recognized as a potential One-Health risk.

### 3.2 Resistomes in production environments of broiler and salmon processing plants

**Figure 6:**
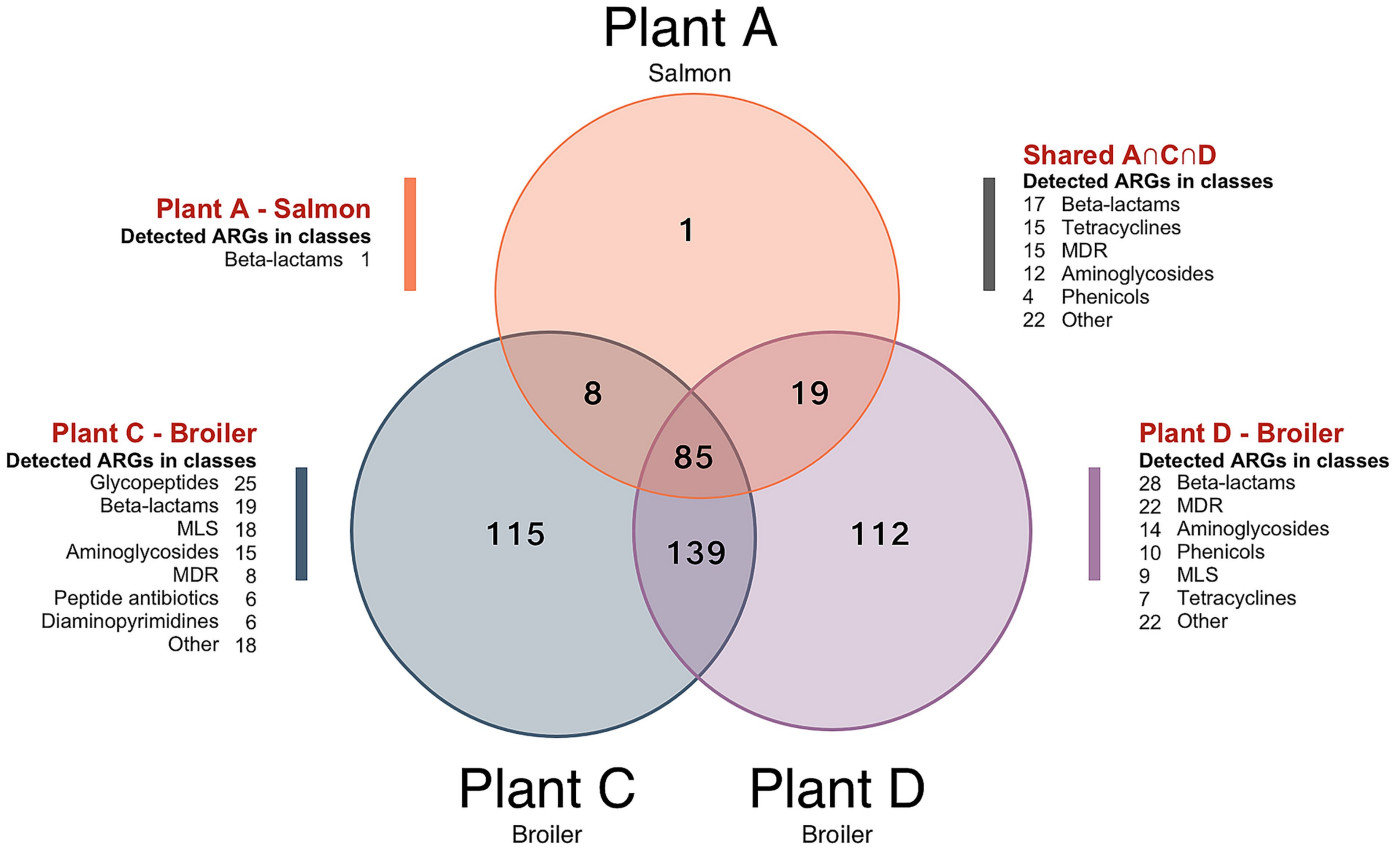
Venn diagram showing the number of antimicrobial resistance genes (ARGs) detected across the combined surface samples from each processing environments of salmon (Plant A) and broiler (Plant C and Plant D). Unique ARGs only detected in one of the plants are displayed by A, C and D, while A ∩ C ∩ D shows the intersection of ARGs found in all three plants. Number of ARGs in different antimicrobial classes are also shown. Abbreviations: MLS for macrolide, lincosamide, streptogramin, and MDR for multidrug resistance.

Surface resistomes on food contact surfaces (FCS) and non-food contact surfaces (NFCS) in processing plants of salmon (Plant A) and broiler (Plant C and D) was investigated. The average number of aligned reads to ARGs was approximately 2.5 × 10^4^ and all samples had reads that aligned to at least one ARG. A total of 479 unique ARGs were detected across all sample types with 113 ARGs detected in the salmon processing Plant A, while 347 and 355 ARGs were detected in the broiler processing Plants C and D, respectively. A core resistome of 85 ARGs present in all three plants was revealed (Figure 6), with beta-lactam and tetracycline resistance genes in the *OXA*- and *tet*-family dominating. More than 99% of the ARGs detected in Plant A were also found in Plant C and D. This was unexpected since the production of salmon differs substantially from the broiler production but is likely explained by the large variety of ARGs detected in the broiler processing Plants C and D. More than 220 genes were also shared among Plant C and D, with approximately 110 unique genes detected in each plant. Differences in primary production, slaughter, processing conditions, and hygienic practices (Reiche et al., 2025) may explain the variation in ARG composition between Plant C and D. Most of the ARGs detected in the Norwegian Plant C belonged to classes of glycopeptides, beta-lactams and MLS, while beta-lactams, MDR and aminoglycoside classes dominated in the Romanian Plant D. A total of 114 high-risk genes were detected across the samples (Table 1), with differences observed between NFCF and FCS. For example, we detected 87 high-risk genes in the transport truck of Plant C, while 37 were detected on the conveyor to the filling machine of broiler meat. Likewise, the number of high-risk ARGs was lower on the post-evisceration chilling belt for whole broilers (FCS) compared to the floors in cutting area (NFCS) in Plant D, with a difference of approximately 30 genes. Nevertheless, the presence of high-risk genes on FCS is concerning since they can be transmitted to food products. MRSA-associated ARGs *mecA, mecR1,* and *mecI* were only detected on NFCS including the transport truck of Plant C. In our previous studies (Reiche et al., 2024, 2025), the bacterial microbiota was sequenced with DNA from the same samples, and *Staphylococcus* was among the most abundant genera in the transport truck. These findings indicate the presence of MRSA, which is uncommon in Norway, where one of the lowest prevalences of MRSA infections in the world has been reported (Di Ruscio et al., 2017). *Acinetobacter, Pseudomonas* and *Enterobacteriaceae* dominated the bacterial microbiota in samples from Plant D, where many high-risk MDR genes were detected. While on the crate washer from Plant A, where the lowest number of high-risk ARGs was detected, *Chryseobacterium* and *Arthrobacter* were among the most abundant genera.

**Table 1:** Overview of high-risk antimicrobial resistance genes (ARGs) detected across the combined surface samples from each processing environment of salmon (Plant A) and broiler (Plant C and Plant D).

Sampling point	Detected high-risk ARGs
Transport truck S1-2 NFCS Plant C—broiler	**Aminoglycosides:** *APH(6)-Id, APH(3″)-Ib, AAC(6′)-Ie-APH(2″)-Ia, APH(3′)-IIIa, kdpE, APH(3′)-Ia, AAC(6′)-Ib7, ANT(4′)-Ib, ANT(6)-Ia* **Beta-lactams:** *mecA, mecR1, mecI* **Fluoroquinolones:** *emrR, emrA, mdtH* **Fosfomycins:** *mdtG* **MLS:** *mphC, mphB, Mef(En2), mphE, ErmB, ErmX, ErmC, ErmT, ErmA* **MDR:** *TolC, MexD, MexC, MexB, H-NS, marA, acrB, acrS, evgS, evgA, acrF, acrE, CRP, mdtE, mdtF, gadX, gadW, smeB, smeS, mtrA, adeJ, smeE, mexW, smeD, smeF, efrA, efrB, efmA, mexQ, msrA, mel, lsaA, lsaE, msrC, oqxB, adeF, ceoB, mdtM, MexF, cpxA, baeR, baeS* **Peptide antibiotics:** *ugd, eptA, pmrF, bacA, yojI* **Sulfonamides:** *sul1, sul2* **Tetracyclines:** *tetX, tetO, tetM, emrY, tetQ, emrK, tet(A), tet(40), tet(K), tet(L), tetW, tet(C), tetA(P), tet32*
Conveyor to filling machine S3-4 FCS Plant C—broiler	**Fluoroquinolones:** *emrA, mdtK* **MLS:** *ErmX* **MDR:** *TolC, MexB, H-NS, ramA, acrB, acrS, acrF, CRP, mdtE, mdtF, gadX, smeB, adeJ, smeE, smeD, efrB, mexQ, oqxB, oqxA, adeF, ceoB, mdtM, MexF, cpxA, baeR, baeS* **Peptide antibiotics:** *pmrF, bacA, yojI* **Phenicols:** *catI* **Tetracyclines:** *tetO, emrY, tet(K), tet(L)*
Floors in cutting area S12-13 NFCS Plant D—broiler	**Aminoglycosides:** *APH(6)-Id, APH(3″)-Ib, AAC(6′)-Ie-APH(2″)-Ia, ANT(2″)-Ia, APH(3′)-IIIa, kdpE, APH(3′)-Ia, AAC(6′)-Ib7, ANT(4′)-Ib, ANT(6)-Ia* **Beta-lactams:** *mecA* **Fluoroquinolones:** *emrR, emrA, mdtH, qacA, mdtK, patA* **Fosfomycins:** *mdtG* **MLS:** *mphA, mphC, mphB, macB, mphE, ErmB, ErmF, ErmX, ErmC, ErmT* **MDR:** *TolC, OprM, mexY, MexC, MexB, MexA, H-NS, marA, acrB, sdiA, evgS, acrF, acrE, CRP, mdtE, mdtF, smeB, smeS, mtrA, adeJ, AxyY, smeE, mexW, smeD, smeF, mexQ, OpmB, mexK, msrA, mel, lsaE, poxtA, lsaC, oqxB, adeF, mexI, mexH, PmpM, mdtM, MexF, MexE, arlS, cpxA, baeR, baeS* **Peptide antibiotics:** *ugd, pmrF, bacA, yojI* **Phenicols:** *catI* **Sulfonamides:** *sul1, sul2* **Tetracyclines:** *tetX, tetO, tetM, emrY, tetQ, emrK, tet(B), tet(A), tet(K), tet(L), tetW, tet(C), tet(D), tetA(P), tet32*
Chilling belt S14-15 FCS Plant D—broiler	**Aminoglycosides:** *APH(6)-Id, APH(3″)-Ib, ANT(2″)-Ia, kdpE, APH(3′)-Ia, AAC(6′)-Ib7* **Fluoroquinolones:** *emrR, emrA, mdtH, mdtK* **Fosfomycins:** *mdtG* **MLS:** *mphA, Mef(En2), mphE* **MDR:** *TolC, MexD, MexC, MexA, H-NS, marA, ramA, acrB, acrS, evgS, evgA, acrF, acrE, CRP, mdtE, mdtF, gadX, smeB, smeS, mtrA, adeJ, smeE, mexW, smeD, smeF, mexQ, mexK, oqxB, oqxA, adeF, mdtM, MexF, cpxA, baeR, baeS* **Peptide antibiotics:** *ugd, eptA, pmrF, bacA* **Sulfonamides:** *sul1, sul2* **Tetracyclines:** *tetX, emrY, tetQ, emrK, tet(B), tet(A), tetW, tet(C), tet(D), tetA(P)*
Crate washer S21-24 NFCS Plant A—salmon	**Aminoglycosides:** *APH(6)-Id, APH(3″)-Ib, AAC(6′)-Ib7* **Fluoroquinolones:** *emrR, emrA* **MLS:** *mphE* **MDR:** *acrB, acrE, CRP, mtrA, adeJ, smeE, mexW, smeD, mexQ, mexK, oqxB, adeF, MexF, cpxA, baeR* **Sulfonamides:** *sul1, sul2* **Tetracyclines:** *tetX, tetM, tet(A), tetW, tet(C)*

Samples include both food contact surfaces (FCS) and non-food contact surfaces (NFCS).

Additionally, we found a total of 24 different ARGs in the transport truck belonging to the *van*-family such as *vanB, vanX, vanRB, vanYB* and *vanHB* conferring resistance to vancomycin. According to the criteria by Zhang et al. (2022) several of these have high host pathogenicity but score low on human accessibility and mobility, and are therefore placed in Q3 group, i.e., not high-risk. Nevertheless, we think these are worth mentioning since vancomycin-resistant *E. faecium* (VRE) is on the World Health Organization’s (WHO) priority list of drug-resistant bacteria most threatening to human health. In our previous study, a variety of *E. faecium* and *E. faecalis* isolates were also detected from the same sample of the transport truck, although antibiotic resistance profiles were not analyzed in these isolates (Reiche et al., 2025). Overall, the transport truck appears to be a hotspot for ARGs, potentially spreading them downstream in the value chain.

As the sampling methodology for the surface samples differed from the methodology used to collect sidestream materials and waste discharges, we cannot compare these results directly. The DNA yield in some of the surface samples was also relatively low and the entry-concentration to the sequencing library differed between samples. Therefore, we have chosen to present the results without abundance data and pooled the samples taken before and after cleaning and disinfection (C&D). A more comprehensive sampling strategy may have provided a larger quantity of samples with sufficient DNA yield to accurately capture differences in ARG prevalence before and after C&D. Despite the incomparability of ARG abundance and diversity, our results indicate that a variety of ARGs were present after C&D in the production environments (Supplementary Table S5). Other studies have also confirmed that C&D fails to remove ARGs completely from food processing environments (Merino et al., 2024; Xiao et al., 2024). Ultimately, the targeted hybrid capture-based sequencing approach (xHYB) we used is a semi-quantitative method lacking the ability to capture absolute ARG abundances such as gene copies per mL. This can be overcome by using digital droplet PCR (ddPCR) or quantitative PCR (qPCR). Furthermore, the efficiency of DNA hybridization may be affected by the presence of organic contaminants and improper purification of DNA prior to library preparation may lead to loss of genes that are more efficiently captured from samples with higher DNA purity. Caution must therefore be taken when comparing data from samples of very different origins. Nevertheless, the method is highly sensitive, and a previous study demonstrated that xHYB appropriately detected ARGs when compared to conventional mDNA-seq (Baba et al., 2023). The authors pointed out that xHYB is limited to the Qiagen AMR-panel, which targets 2,786, but noted that these cover more than 93% of the ARGs in the NCBI/ResFinder database. Altogether, our findings show that salmon and broiler processing environments serve as reservoir of ARGs, with more studies needed on the absolute abundances to assess the risk and impact on AMR spread.

### 3.3 Identification of bacterial isolates from waste discharges and sidestream materials

**Figure 7:**
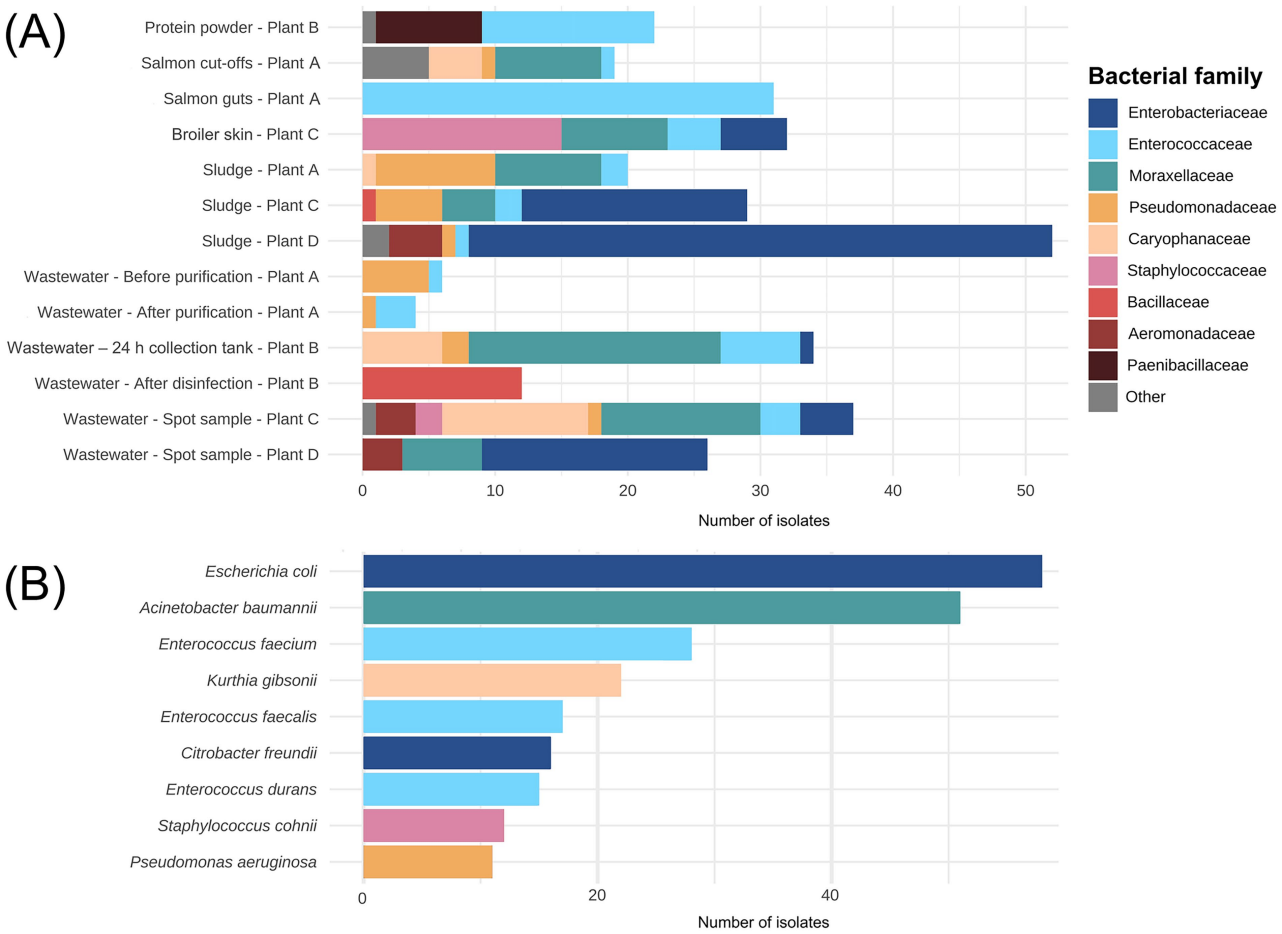
**(A)** Number of bacterial isolates (n = 324) assigned to family level isolated from sidestream materials and waste discharges from processing plants of salmon (Plant A), sidestream materials (Plant B) and broiler (Plant C and Plant D). The fish meal sample from Plant B is not shown since no bacterial isolates were detected. **(B)** The nine most identified species among the 324 isolates.

A total of 324 bacterial isolates were collected from the sidestream materials and waste discharges across salmon and broiler processing Plants A, C, and D, and the sidestream material processing Plant B. Among the isolates, we found that *Enterobacteriaceae* (27%), *Enterococcaceae* (21%) and *Moraxellaceae* (20%) were the most prevalent families (Figure 7A). More than 99% of the *Enterobacteriaceae* isolates originated from the broiler processing Plants C and D. At lower taxonomic levels, *E. coli* (15%), *A. baumannii* (15%) and *E. faecium* (15%) were the most detected species (Figure 7B). The latter was mainly found in salmon sidestream materials, while numerous of isolates in the *Staphylococcaceae* family were found on the broiler skin residual raw material including *S. chromogenes* and *S. cohnii.* Sludge samples from broiler processing Plants C and D harbored most of the *E. coli* and *Citrobacter freundii* isolates we detected. Interestingly, only spore-forming bacteria such as *Bacillus cereus* and *B. licheniformis* were found in the wastewater after disinfection by hypochlorite (Plant B) where the largest number of ARGs were detected (330). Suggesting that disinfection inactivated the vegetive bacteria that we found prior to disinfection but failed to remove ARGs. *P. aeruginosa* was detected in salmon cut-offs from Plant A, in addition to wastewater from Plant A, B and C and sludge from Plant C. Overall, we found many ESKAPEE bacteria among the isolates excluding *S. aureus* and *K. pneumoniae* (details in Supplementary Table S1; Supplementary Figures S1, S2).

### 3.4 Antibiotic susceptibility in isolates from waste discharges and sidestream materials

**Figure 8:**
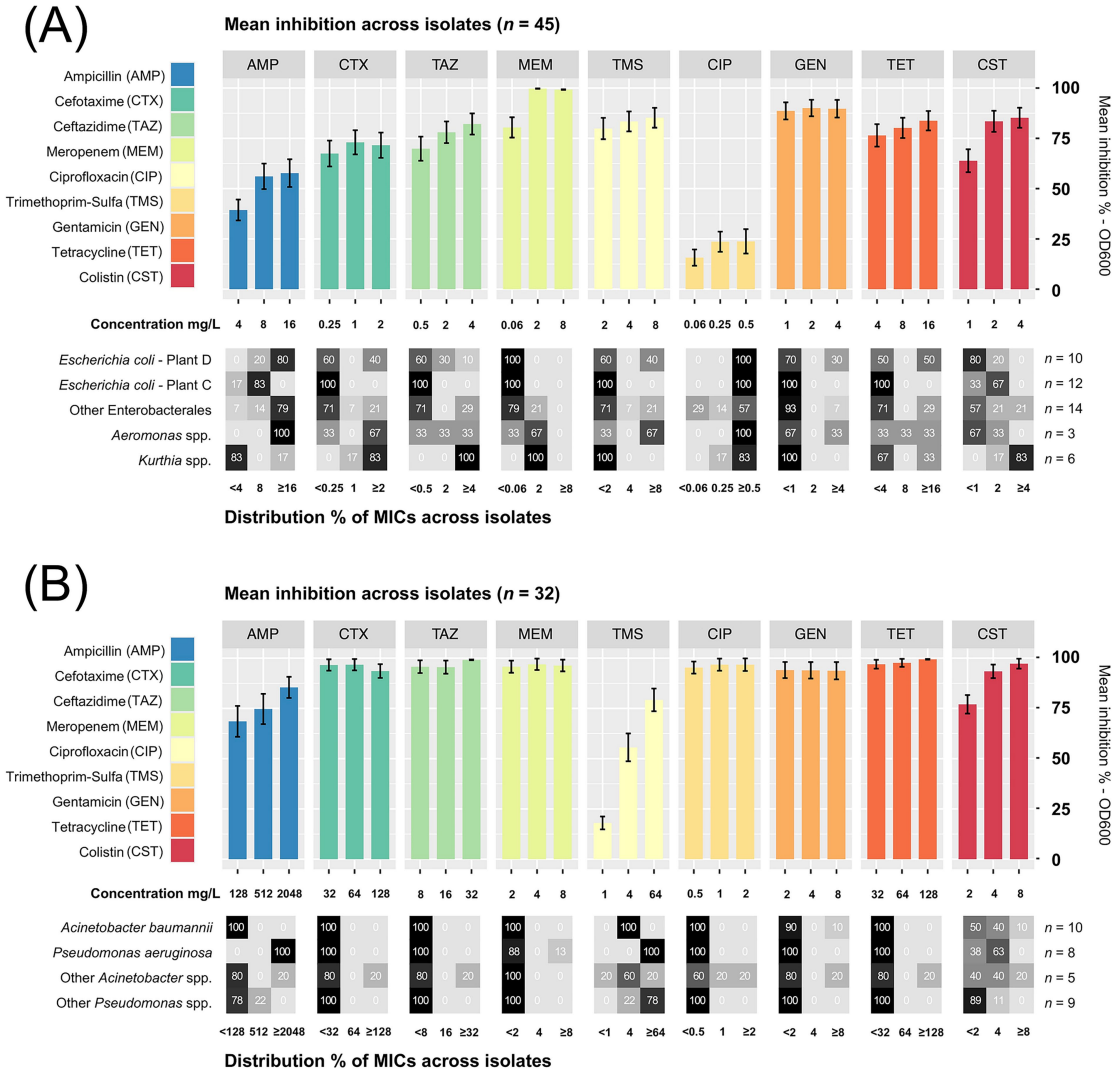
**(A)** Mean inhibition % across isolates of Enterobacterales, *Aeromonas* spp. and *Kurthia* spp. (*n* = 45) tested against nine di!erent antibiotics from seven classes. **(B)** *Acinetobacter* spp. and *Pseudomonas* spp. (*n* = 32) were tested against the same antibiotics using higher concentrations. The distribution % of minimum inhibitory concentrations (MICs) for groups of isolates are shown below each bar plot (detailed list in Supplementary Table S10). Error bars show the standard error of the mean (SEM).

**Figure 9:**
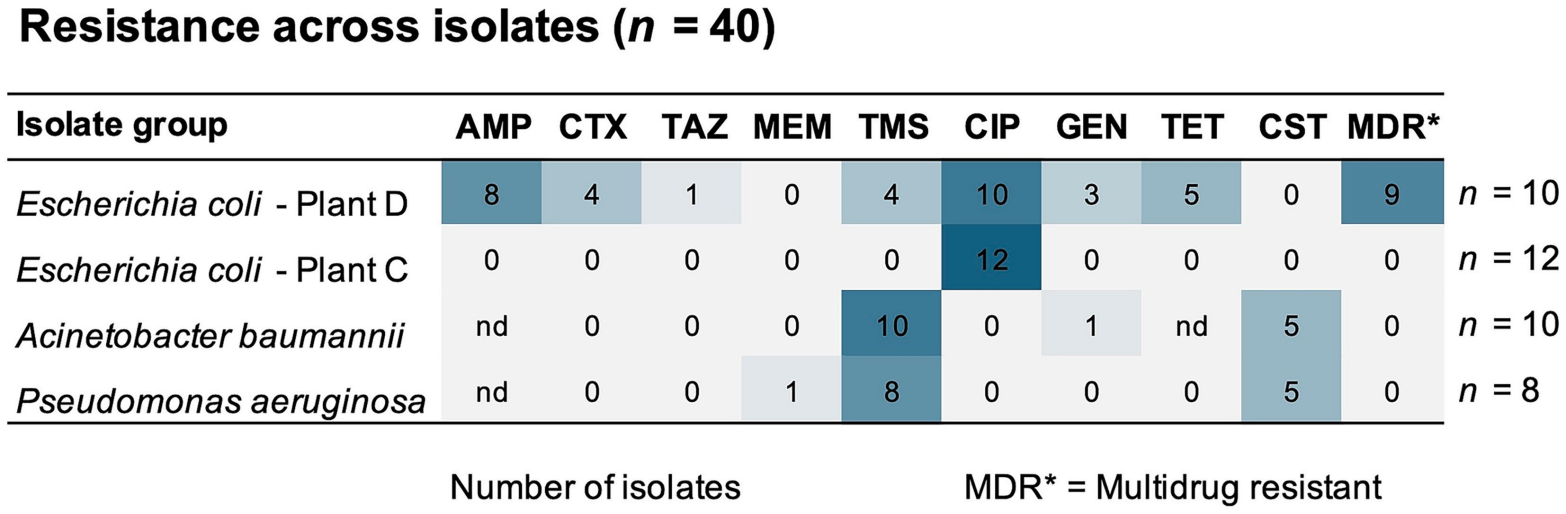
Number of isolates (*n* = 40) with resistance to antibiotics from seven different classes. The selection includes *E. coli* isolates from broiler processing plants (Plant C and D) and isolates of *A. baumannii*, and *P. aeruginosa* detected across all processing plants. nd indicates that no data for cut-off values was available. AMP, ampicillin; CTX, cefotaxime; TAZ, ceftazidime; TMS, trimethoprim-sulfamethoxazole; CIP, ciprofloxacin; GEN, gentamicin; TET, tetracycline; CST, colistin.

A high-throughput screening (HTS) assay was performed to determine the antibiotic susceptibility in two selected groups of bacterial isolates: (1) Enterobacterales, *Aeromonas* and *Kurthia* isolates (*n* = 45), and (2) *Pseudomonas* and *Acinetobacter* isolates (*n* = 32). Each group was tested against nine antibiotics using lower concentrations for the former (1) and higher for the latter (2). Among isolates in selection (1), we found generally high CIP MICs (≥0.5 mg/L) with an overall low inhibition percentage (Figure 8A). This was expected since most of these bacteria were isolated using selective media containing CIP. All *E. coli* isolates from broiler sidestreams and waste discharges were confirmed as quinolone resistant (QREC) with distinct differences between isolates from Plant C and D. For six out of nine antibiotics, the MICs were lower in *E. coli* from Plant C (Norway) compared to *E. coli* from Plant D (Romania) of which all except one isolate was MDR (Figure 9).

Among these nine MDR isolates, four were also confirmed as ESBL-producers, while none of the *E. coli* from Plant C tested positive for ESBL production (detailed MIC-lists in Supplementary Tables S6–8). A follow-up analysis using a broader range of CIP concentrations revealed additional differences. In *E. coli* from Plant D, CIP MICs ranged between 8 and >16 mg/L, which is almost 200 times higher than the epidemiological cut-off value (ECOFF) of 0.06 mg/L. Far lower MICs were found in *E. coli* from Plant C, corresponding to ≤1 mg/L CIP. A previous study has shown that quinolone resistance in *E. coli* originating from healthy broilers in Norway is low (<10%) (Kaspersen et al., 2018), while a similar study from Romania found that 73% of *E. coli* from Romanian broilers were quinolone resistant, with MIC values ranging between 0.015–8 mg/L CIP (Rus et al., 2025). Nevertheless, both studies used non-selective methods targeting *E. coli*, where the occurrence of QREC is analyzed in a random pool of *E. coli* isolates.

Moreover, we observed high ampicillin MICs in *P. aeruginosa* corresponding to ≥2048 mg/L (Figure 8B). One *P. aeruginosa* isolate, found in wastewater (Plant C), was also carbapenem resistant with an MIC of ≥8 mg/L meropenem. All *P. aeruginosa* and *A. baumannii* isolates were also resistant to trimethoprim-sulfamethoxazole, and half were colistin resistant (MIC >2 mg/L) (Figure 9). These were isolated from wastewater and sludge in Plant A, B, and C. Nevertheless, *P. aeruginosa* and *A. baumannii* were fully susceptible to cefotaxime, ceftazidime, ciprofloxacin and tetracycline. Since environmental isolates were tested in this study, we applied ECOFF values to distinguish between susceptibility and resistance. However, the resistance level could not be determined for several species due to the lack of ECOFF values. An overview of susceptibility and resistance based on clinical breakpoints is available in Supplementary Figure S3. Based on these breakpoints, a few *C. freundii* isolates detected in waste discharges (Plant D and B), *E. kobei* (Plant C), *Raoultella planticola* and *Aeromonas veronii* (Plant D) were also MDR.

Antibiotic susceptibility testing of *Enterococcus* isolates (*n* = 67) revealed an overall low prevalence of resistance, and none of the isolates were MDR (Table 2). *E. faecalis* isolates were susceptible to all twelve antibiotics except one isolate with tetracycline resistance from sludge (Plant D). Full susceptibility in *E. faecium* and other *Enterococcus* spp. was observed for eight and nine antibiotics, respectively. The highest percentage of resistance was observed in *E. faecium* isolates towards erythromycin (66%), Quinupristin/daldopristin (24%) and tetracycline (21%). Most of the erythromycin resistant isolates (63%) originated from protein powder (Plant B) and salmon guts (Plant A) (21%). Tetracycline resistant isolates were found both in wastewater (Plant C) and salmon guts (Plant A). One *E. faecium* isolate, retrieved from the wastewater 24 h collection tank (Plant B), was resistant towards vancomycin (VRE). A significant increase in the prevalence of clinical VRE has been reported in Europe during 2016–2020 (WHO, 2022). However, the clinical prevalence in Norway remains low, with an annual rate of 1.6 per 100,000 individuals reported in 2023 (NORM/NORM-VET, 2023). Altogether, we consider the AMR risk of the *Enterococcus*-isolates as low since most of the strains were fully susceptible towards clinically important antibiotics, including ampicillin, linezolid, vancomycin and daptomycin which are used to treat *Enterococcus* infections.

**Table 2:** Resistance % and minimum inhibitory concentration (MIC) range across *Enterococcus* isolates (*n* = 67) tested towards twelve antibiotics (Sensititre susceptibility assay).

Antibiotic	Concentration range (μg/mL)	Resistance (%) and MIC range					
		*E. faecium* (*n* = 29)		*E. faecalis* (*n* = 17)		*Enterococcus* spp. (*n* = 21)	
Ampicillin	0.5–64	0	[<0.5–2]	0	[<0.5–1]	0	[<0.5]
Gentamycin	8–1,024	0	[<8–32]	0	[16–64]	0	[<8]
Erythromycin	1–128	**66**	**[<1–32]**	0	[<1–4]	**10**	**[<1–16]**
Tetracycline	1–128	**21**	**[<1–>128]**	**6**	**[<1–64]**	**10**	**[<1–64]**
Tigecycline	0.03–4	0	[<0.03–0.12]	0	[<0.03–0.06]	0	[0.06–0.12]
Ciprofloxacin	0.12–16	0	[<0.12–8]	0	[0.5–2]	0	[0.25–2]
Vancomycin	1–128	**3**	**[<1–8]**	0	[<1–2]	0	[<1–4]
Teicoplanin	0.5–64	0	[<0.5]	0	[<0.5]	0	[<0.5]
SYN*	0.5–64	**24**	**[<0.5–4]**	0	[2–8]	0	[<0.5–4]
Daptomycin	0.25–32	0	[1–8]	0	[0.5–4]	**5**	**[0.5–8]**
Linezolid	0.5–64	0	[<0.5–2]	0	[1–2]	0	[1–2]
Chloramphenicol	4–128	0	[<4–8]	0	[<4–8]	0	[<4–8]

*SYN (Synercid) Quinupristin/daldopristin.

Resistance % above zero in bold.

### 3.5 Analysis of whole genome sequences of *Escherichia coli*

*Escherichia coli* with high levels of phenotypic resistance from broiler processing Plant D in Romania (*n* = 7) and Plant C in Norway (*n* = 3) were whole genome sequenced to investigate their genotypes. Raw reads were assembled into draft genomes with a total size of 5.0–5.5 Mb, distributed onto 318–864 contigs and an average sequencing depth of 22.6–38.8x (assembly statistics available in Supplementary Table S10).

Among MDR *E. coli* from Plant D, we found multiple ARGs, plasmids and mobile genetic elements (MGEs) (Table 3). All ESBL-phenotypes harbored the *bla_CTX-M-1_* gene, with isolate DES10.1 carrying an insertion sequence (ISEc9) located on the same contig. The *bla_CTX-M_* gene is usually plasmid encoded and spread through conjugation, but a recent study demonstrated that ISEc9 can facilitate transposition of *bla_CTX-M_* after uptake of DNA by transformation (Domingues et al., 2025). In isolate DQS6.1, we detected twelve ARGs with a clear connection to its phenotype. Aminoglycoside resistance genes such as *aac(3)-IV, aph(4)-Ia, ant(3″)-Ia,* and *aadA1* likely explain resistance to gentamicin, which is in the class of aminoglycosides. Whereas resistance towards tetracycline, trimethoprim-sulfamethoxazole and ciprofloxacin is associated with *tet(A), sul2* and *qnrS1*, respectively. Isolate DQS6.1 additionally carried several co-located ARGs and MGEs such as *tet(A)* co-located with a transposon (Tn5403), and *sul2* co-located with a plasmid (IncI1) and insertion sequence (ISSbo1). IncI1 is one of the most common type of plasmids among *Enterobacteriaceae* and play an important role in the transmission and spread of AMR worldwide (Carattoli et al., 2021). Moreover, we found *qnrS1* and *bla_TEM-1B_* co-located with plasmid IncX1 and MGEs (Tn2 and ISKpn19) in isolate DQS8.1. These results clearly highlight the potential mobility of the ARGs present among *E. coli* isolated from waste discharges in the Romanian broiler processing Plant D. A previous study investigating *E. coli* from Romanian chicken meat found similar ARGs using multiplex PCR. Among the isolates, 53% harbored *tetA*, followed by *bla_TEM_* (37%), *sul1* (27%), *aadA1* (23%), *bla_CTX_* (17%), *qnrA* (17%) and *aac* (10%) (Brătfelan et al., 2023). The occurrence of these ARGs may be a result of the extensive use of antibiotics for food-producing animals in Romania. However, Brătfelan et al. (2023) also reported that data regarding the use of antimicrobials in broiler farms specifically is not published.

The number of ARGs among the three *E. coli* isolates from Plant C was lower, with only one isolate (CQS1.1) harboring antibiotic related ARGs. These were associated with aminoglycoside resistance (*aph(6)-Id* and *aph(3″)-Ib*) but not expressed phenotypically. The disinfectant resistance gene *sitABCD* conferring resistance to hydrogen peroxide was found among all *E. coli* from Plant C and D (list of locus_tags for ARGs available in Supplementary Table S11). Furthermore, we found chromosomal mutations mediating AMR among all isolates. Mutations in *gyrA, gyrB, parE* and *parC* represent major fluoroquinolone resistance mechanisms (Slettemeås et al., 2024) and likely contributed to the isolates’ high resistance to CIP. While *E. coli* from Plant D exhibited two mutations in *gyrA* (p. S83L and p. D87N), *E. coli* from Plant C only had one (p. S83L). The additional mutation may have led to a higher CIP MIC in isolates from Plant D. In addition to the presented chromosomal mutations mediating AMR in Table 3, all isolates besides DQS6.1 and CQB10.1 had also mutations in *16S-rrs, rrsC* and *rrsH* associated with aminoglycoside resistance.

**Table 3:** Antimicrobial resistance (AMR) phenotypes and genotypes of ten *E. coli* isolates.

Source	ID	AMR phenotype	CIP MIC	Linked ARGs (plasmid/MGE)	Unlinked ARGs	Chromosomal mutations mediating AMR
Plant D	DQS6.1	CIP, TMS, GEN, TET, MDR	>16	***tet(A)*** (Tn5403); **sul2** (IncI1 / ISSbo1); ***aac(3)-IV, aph(4)-Ia*** (ISEc37, ISEc59)	*dfrA15, ant(3″)-Ia, aadA1, cmlA1, sul3, floR, qnrS1, sitABCD*	**Fluoroquinolone resistance:** *gyrA(p. S83L), gyrA(p. D87N), gyrB, parE, parC(p. S80I)* **Other:** *pmrA, pmrB, folP, 23S rRNA, rpoB, ampC*-promoter
	DQS3.1	CIP, nd*	>16	***aac(3)-IV, aph(4)-Ia*** (ISEc59); **sul2** (ISVsa3); ***bla***_***TEM-1C***_ (Tn2)	*tet(A), sitABCD*	
	DES5.1	CIP, AMP, CTX, ESBL, MDR	16	none	*bla_CTX-M-1_, sitABCD*	
	DES10.1	CIP, AMP, CTX, ESBL, MDR	8	***bla***_***CTX-M-1***_ (ISEc9)	*sitABCD*	
	DQW5.1	CIP, AMP, CTX, TAZ, ESBL, MDR	8	none	*bla_CTX-M-1_, sitABCD*	
	DES7.1	CIP, AMP, CTX, ESBL, MDR	8	none	*bla_CTX-M-1_, sitABCD*	
	DQS8.1	CIP, AMP, TET, MDR	>16	***tet(A)*** (IS30), ***qnrS1, bla***_***TEM-1B***_ (IncX1, Tn2, ISKpn19)	*sitABCD*	
Plant C	CQW2.2	CIP	1	none	*sitABCD*	**Fluoroquinolone resistance:** *gyrA(p. S83L), gyrB, parE, parC(p. S80I)* **Other:** *pmrA, pmrB, folP, 23S rRNA, rpoB, ampC*-promoter
	CQB10.1	CIP	1	***sitABCD*** (ISKox3)	none	
	CQS1.1	CIP	1	none	*aph(6)-Id, aph(3″)-Ib, sitABCD*	

Linked antimicrobial resistance genes (ARGs) in bold are co-located on the same contig with plasmids (Inc) or MGEs (mobile genetic elements) including insertion sequences (IS) and transposons (Tn). ARGs on contigs without plasmids or MGEs are shown as unlinked. The strains were isolated from waste discharges from broiler processing plants (Plant C and D), except for CQB10.1, which was isolated from broiler skin (Plant C).

*nd indicates no data, i.e., the isolate was not included in the HTS susceptibility tests.

CIP, ciprofloxacin; AMP, ampicillin; CTX, cefotaxime; TAZ, ceftazidime; TMS, trimethoprim-sulfamethoxazole; GEN, gentamicin; TET, tetracycline; ESBL, extended spectrum beta-lactamase producing; MDR, multidrug resistant.

Interestingly, most of the ARGs in the *E. coli* genomes were also detected in our resistome sequencing-based analysis with some of the detected genes in a different version. For example, in sludge from Plant D, we detected *aac(3)-IV, aph(4)-Ia, sul2, tet(A), bla_CTX-M_, bla_TEM_,* all of which are present in the *E. coli* genomes isolated from the same sludge. Only *qnrS1* was not detected in the resistome, but a different version of the gene was found (*qnrS6*). Matching ARGs in resistomes and whole genomes were also observed in wastewater from Plant C and D. The disinfectant-resistance gene *sitABCD* was not detected in resistomes since the AMR panel lacks probes capturing this gene. Altogether, this was expected since the *E. coli* strains were isolated from the same samples, but it also confirms the sensitivity and performance of the targeted hybrid capture-based sequencing approach.

Moreover, *E. coli* isolates were assigned to six serotypes, five multilocus sequence types (MLSTs) and eight core genome (cg) MLSTs (Table 4). ST 162 was most prevalent among isolates from Plant D and has been previously found among avian pathogenic *E. coli* (APEC) isolated from diseased chicken in the Czech Republic (Papouskova et al., 2020). Among the isolates from Plant C, CQW2.2 shared the same serotype and MLST as DQS8.1 from Plant D, although a large difference was observed in the genotypic and phenotypic AMR profile between these isolates. Whereas *E. coli* CQB10.1 was assigned to ST 155, which has been previously identified among QREC isolated from urinary tract infection and bacteremia cases in Norway (Slettemeås et al., 2024). Furthermore, we found that the following isolates shared the same serotype and cgMLST profiles: DES5.1 and DES10.1 (cgST 187,026), and DQW5.1 and DES7.1 (cgST 207,265), although they were not identical. A single nucleotide polymorphism (SNP) analysis revealed 13 SNP differences between DES5.1 and DES10.1, and 8 SNP differences between DQW5.1 and DES7.1 with a sequencing depth threshold of >10×. However, the sequencing depth in our data provides limited resolution for accurately mapping these differences. Higher-depth whole genome sequencing (e.g., ≥100×) would be required for a more precise comparative analysis.

A variety of virulence genes were also detected in all ten QREC isolates (Table 4). Genes encoding hemolysins (*hlyE* and *hlyF*), adhesins (*fdeC* and *hra*), fimbriae (*fimH*) and iron uptake systems (*iroN* and *iucC*) were found among most of the isolates. All isolates from Plant C also harbored the *astA* gene encoding heat-stable enterotoxins, while a type III secretion system (T3SS) was found in one isolate (CQS1.1). With an overall high pathogen potential (>0.93), the isolates are likely pathogenic to humans (complete list of all virulence genes and pathogen potential in Supplementary Figure S4; Supplementary Table S9).

**Table 4:** Overview of genotypic characteristics including serotype, MLST (multilocus sequence type) and core genome MLST (cgMLST) of ten *E. coli* isolates.

Source	ID	Serotype	MLST	cg MLST	Virulence genes		
					Secretion system^1^ and toxins^2^	Adhesins^3^ and fimbriae^4^	Iron uptake^5^ and capsules^6^
Plant D	DQS6.1	O9:H11	ST 162	cgST 39961	*^2^hlyE, hlyF*	*^3^fdeC, hra, tia, ^4^fimH, papC*	*^4^iucC, iutA, sit*
	DQS3.1	O131:H12	ST 359	cgST 25122	*^2^hlyE, hlyF, tsh*	*^3^fdeC, hra, ^4^etsC, fimH*	*^4^iroN, iucC, iutA, sitA*
	DES5.1	O153:H19	ST 162	cgST 187026	*^2^hlyE, hlyF, tsh*	*^3^fdeC, hra, ^4^etsC, fimH, papC*	*^4^iroN, iucC, iutA, sitA*
	DES10.1						
	DQW5.1	O153:H19	ST 162	cgST 207265			
	DES7.1						
	DQS8.1	O83:H42	ST 1485	cgST 188418	*^2^astA, hlyE, tsh*	*^3^fdeC, hra, tia, iha, ^4^fimH*	*^4^chuA, iroN, iucC, iutA, sitA, ^5^kpsE, kpsMII_K4*
Plant C	CQW2.2	O83:H42	ST 1485	cgST 166873	*^2^astA, hlyE, hlyF*	*^3^fdeC, hra, tia, iha, AslA, ^4^etsC, fimH*	*^4^chuA, ireA, iroN, iucC, iutA, sitA, ^5^kpsE, kpsMII_K5*
	CQB10.1	O78:H17	ST 155	cgST 117915	*^2^astA, hlyE, hlyF*	*^3^fdeC, iha, tia, ^4^fimH, papC*	*^4^sitA, irp2, iucC, iutA, fyuA*
	CQS1.1	O26:H34	ST 752	cgST 130951	*^1^eae-, tir, espA, espB, nleA, nleB, cif, ^2^astA, hlyE, hlyF, tsh*	*^3^fdeC, tia, ^4^etsC, fimH*	*^4^iroN, iucC, iutA, sitA*

Superscript numbers 1 to 6 for secretion system^1^, toxins^2^, adhesins^3^, fimbriae^4^, iron uptake^5^ and capsules^6^.

The strains were isolated from waste discharges from broiler processing plants (Plant C and D), except for CQB10.1, which was isolated from broiler skin (Plant C). Full list of virulence genes is found in Supplemental Figure S4.

### 3.6 Development of disinfectant tolerance among *Pseudomonas*-isolates

In addition to the investigation focused on the occurrence of AMR bacteria and genes, we conducted an exploratory analysis to examine whether bacterial isolates develop disinfectant tolerance in the presence of increasing concentrations of disinfectants commonly used in the food industry. The results provide insight into the role of non-antibiotic drivers such as disinfectants in the development of bacterial tolerance and resistance.

**Figure 10:**
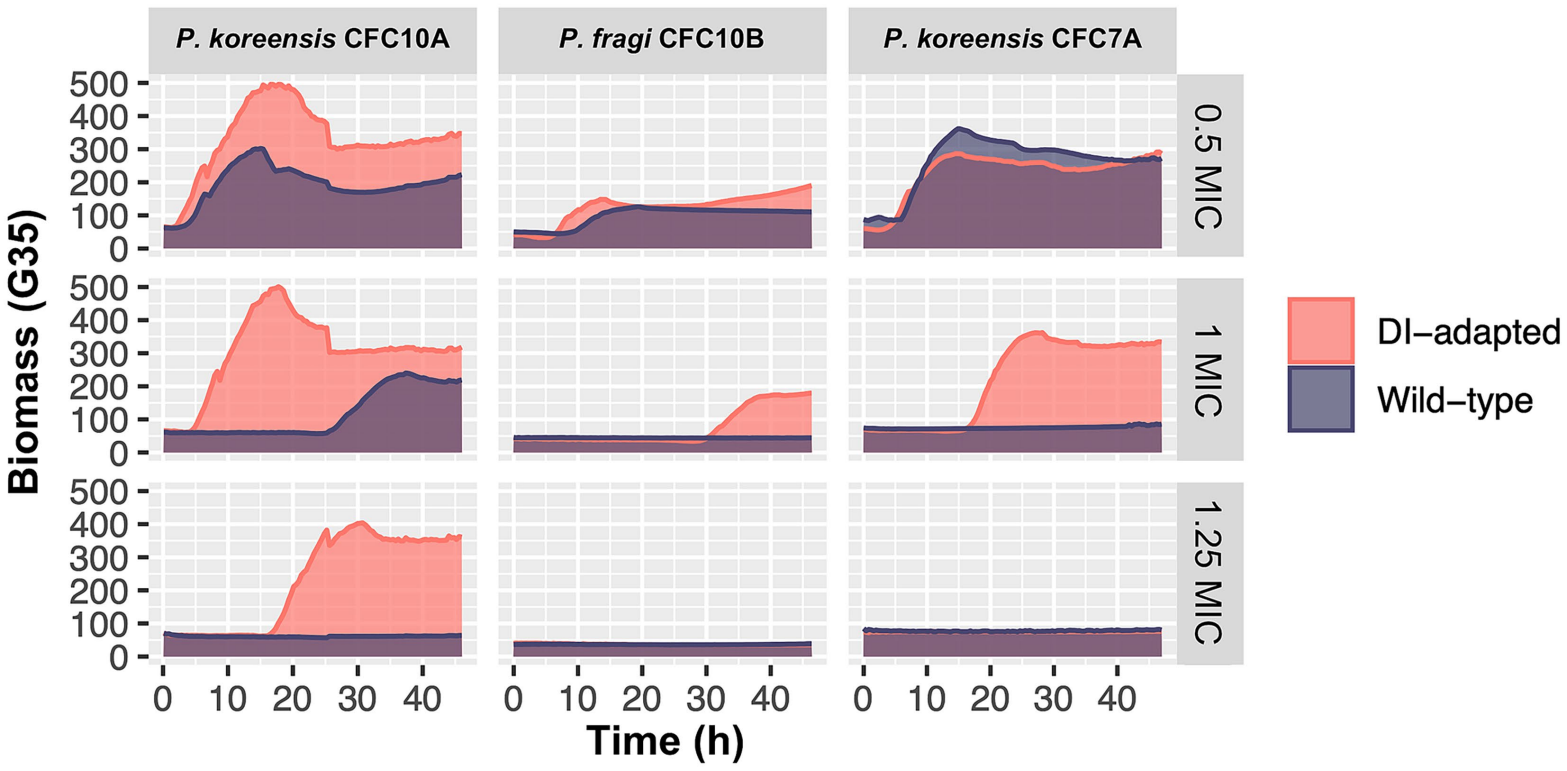
Growth comparison between disinfectant (DI) -adapted and wild-type *Pseudomonas* strains in the presence of sub-minimum inhibitory concentration (0.5 MIC), equal to-MIC (1) and above-MIC (1.25) concentrations of the disinfectant Aqua Des Foam PAA. Isolates are classified in the *P. fluorescens* group: *P. koreensis* CFC10A-1-1, *P. koreensis* CFC7A-4-2 and *P. fragi* CFC10B-1-1. The first and second were isolated from a crate washer and a drain in Plant A (salmon), respectively, while the latter was isolated from a brining vessel in Plant C (broiler). Each growth curve represents the mean of triplicates, based on 2–3 individual biomass (gain G = 35) readings per hour.

A selection of sixteen isolates, primarily in the group of *P. fluorescens,* were exposed to sub-inhibitory concentrations of the peracetic acid and hydrogen peroxide-based disinfectant Aqua Des Foam PAA (ADF). Starting at 1/8 of the isolates’ MIC, the concentrations were increased after each incubation cycle until the isolates were fully inhibited. After nine incubation cycles corresponding to more than 220 h of disinfectant exposure, three out of sixteen isolates showed disinfectant-adaptation (Supplementary Figures S5–7), confirmed by a subsequential MIC test comparing the disinfectant-adapted strains to the original wild-type strains (Figure 10). The results showed that both the wild-type and disinfectant-adapted strains were growing at 0.5 MIC (ADF) as expected. At concentrations equal to 1 MIC (ADF), only the disinfectant-adapted strains (*P. koreensis* CFC7A-4-2 and *P. fragi* CFC10B-1-1) were growing, while the wild-type strains were inhibited. Nevertheless, none of these strains tolerated 1.25 MIC (ADF) which indicates that the development of tolerance was minor. For *P. koreensis* CFC10A-1-1, we observed that both the disinfectant-adapted and wild-type strains were growing at 1 MIC (ADF) with a long lag-time for the latter. At 1.25 MIC (ADF), the wild-type strains were fully inhibited, while the disinfectant-adapted strain was growing after approximately 15 h of incubation.

Furthermore, both the wild-type and disinfectant-adapted strains were tested against a panel of nine different antibiotics in three concentrations. The results showed that antibiotic susceptibility was not decreased in the disinfectant-adapted strains compared to the wild-type strains (MICs in Supplementary Table S6). In a previous study investigating cross-resistance, *E. coli* strains were exposed to sub-inhibitory concentrations of different disinfectants for 25 days prior to antibiotic MIC testing (Merchel et al., 2021). The authors reported no cross-resistance to antibiotics for peracetic acid and only a low impact on susceptibility for hydrogen peroxide. Whereas Benzalkonium chloride, glutaraldehyde, and chlorhexidine had the highest impact on antibiotic susceptibility. In our study, small decreases in antibiotic susceptibility could not be detected since we used two-fold concentrations of antibiotics. A narrower concentrations range may have captured smaller differences. Overall, our results indicate that isolates in the *P. fluorescens* group develop low-level disinfectant tolerance upon prolonged exposure towards sub-inhibitory disinfectant concentrations with more studies needed to confirm the effect on antibiotic susceptibility.

## 4 Conclusion

From a One-Health perspective, the entire food production value chain, including feed, residual raw materials, wastewater and sludge, should be considered with respect to food safety and spread of AMR. New pathways for AMR transmission have emerged, as the waste-flow in food production is altered due to growing focus on circular economy and increased resource exploitation. Mapping reservoirs of AMR across value chains and their environmental dissemination pathways is therefore important for limiting the spread and impact of AMR.

A low prevalence of AMR bacteria was detected in sidestream materials and waste discharges of salmon and broiler, including a few ESBL-producing Enterobacterales, one carbapenem-resistant *P. aeruginosa* (CRPA) and one vancomycin-resistant *Enterococcus* (VRE). However, several quinolone-resistant *E. coli* (QREC) were detected in waste discharges from two broiler processing plants. MDR *E. coli* harboring multiple ARGs, MGEs, plasmids and virulence genes were found in one of the broiler processing plants.

Additionally, a diverse range of ARGs were detected across the sample types. The most frequently detected genes were associated with beta-lactam, tetracycline and aminoglycoside resistance, which are widely used antibiotics on a global scale. High-risk genes were detected on FCS in broiler production environments. This emphasizes that food safety-related consumer awareness is important, since broiler meat products may be contaminated by harmful ARGs, which could spread in household-kitchen environments if meat products are handled incorrectly. Nevertheless, the number of high-risk genes was far lower on FCS compared to NFCS as expected. The overall largest number of ARGs was detected in waste discharges, including high-risk MDR genes such as *TolC*, *OqxB* and *adeJ* which confer resistance towards numerous antibiotics. Among the sidestream materials, salmon cut-offs had the highest number of ARGs of which many have been previously found in sushi-related *Aeromonas* spp. Various ARGs were also detected in salmon protein powder, which indicates that feed ingredients may act as an AMR reservoir.

Ultimately, these results highlight the potential of AMR spread to the environment and into new circular production systems and could be recognized as a potential One-Health risk. Maintaining high quality and food safety standards for residual raw materials is important to control the bacterial burden, minimize the prevalence of AMR bacteria and genes, and to enable their safe use in the production of feed ingredients. Given the relatively small sample size for each sample type in this study, further investigations are needed to confirm our results and to assess the risk of ARG dissemination more accurately. Future research is also needed to develop effective treatment processes that eliminate ARGs from wastewater. Such technology is important to prevent AMR pollution and reduce the risk of releasing wastewater to the surrounding environment such as rivers, fjords and oceans.

## Data Availability

The Whole Genome Shotgun project has been deposited at DDBJ/ENA/GenBank under the accessions JBODOH000000000, JBODOI000000000, JBODOJ000000000, JBODOK000000000, JBODOL000000000, JBODOM000000000, JBODON000000000, JBODOO000000000, JBPDWN000000000, and JBPDWO000000000. The version described in this paper is version JBODOH010000000, JBODOI010000000, JBODOJ010000000, JBODOK010000000, JBODOL010000000, JBODOM010000000, JBODON010000000, JBODOO010000000, JBPDWN010000000, and JBPDWO010000000. The raw sequencing reads from this study have been deposited in the NCBI Sequence Read Archive (SRA) under BioProjects PRJNA1266381 (whole genome sequences, https://www.ncbi.nlm.nih.gov/bioproject/1266381) and PRJNA1266413 (AMR gene resistomes, https://www.ncbi.nlm.nih.gov/bioproject/1266413). 16S rRNA Sanger sequences of isolates are available in Supplementary Table S1.
