# Supplementary material for: Antimicrobial resistance reservoirs in salmon and broiler processing environments, sidestreams, and waste discharges
Source: Front Microbiol. 2025 Sep 16;16:1662113. doi: 10.3389/fmicb.2025.1662113 (PMC12479563; doi:10.3389/fmicb.2025.1662113)
Supplement: Supplementary file 1 [file Data_Sheet_1.PDF]

Supplementary Material: Figures

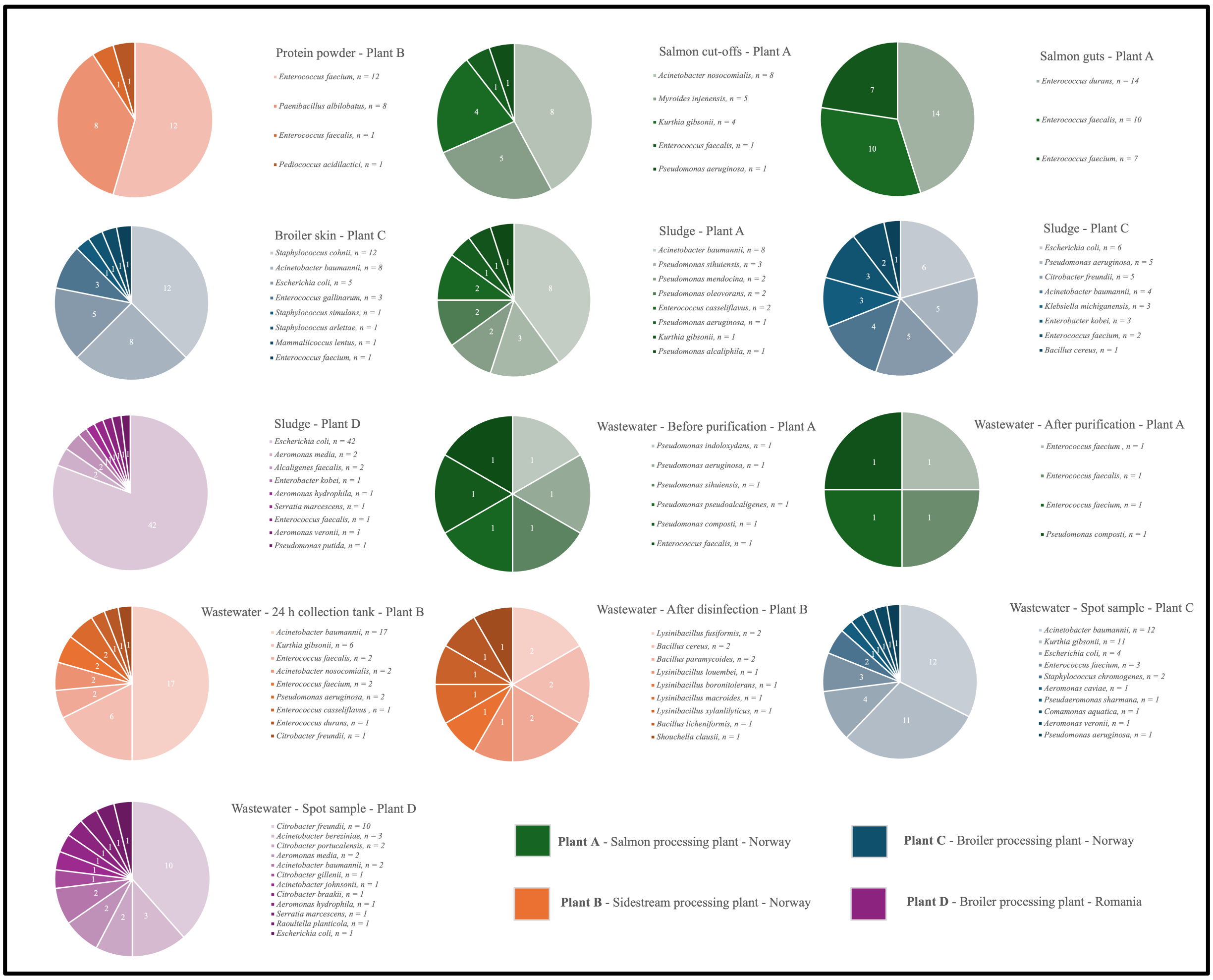

Supplementary Figure S1 Overview of identified species (n = 324) by 16S rRNA sequencing in sample types of residual raw materials, feed ingredients, wastewater and sludge collected from four processing plants. Sample S11 fish meal is excluded since no bacterial isolates were detected.

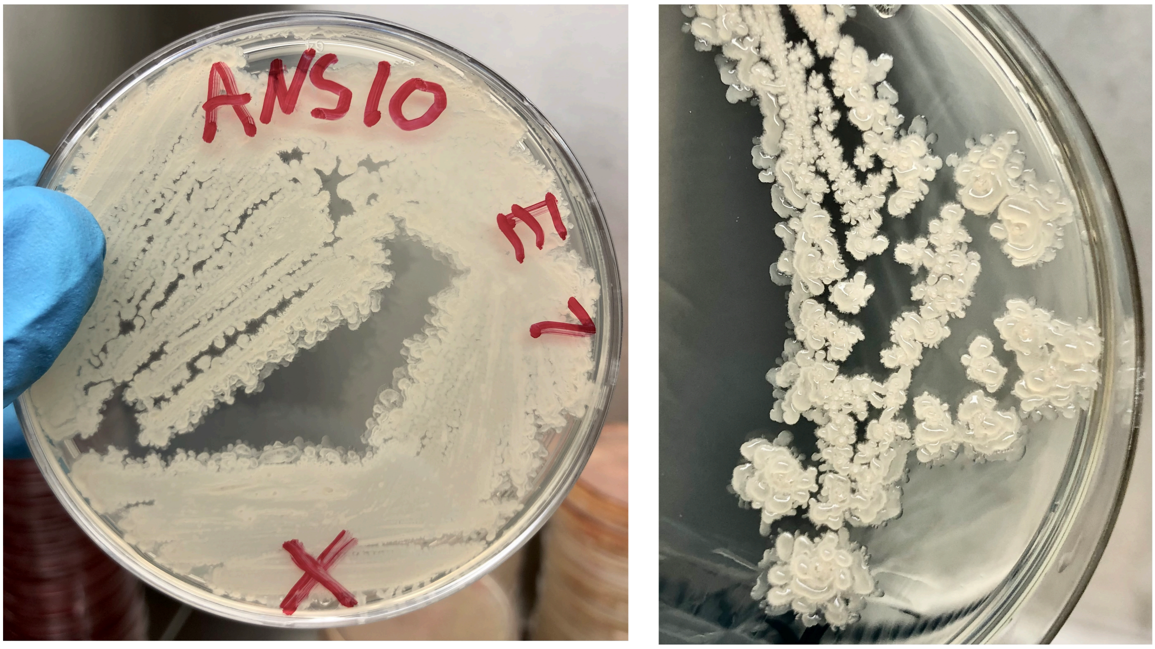

Supplementary Figure S2 Unique colony morphology by isolate BEWA10.1 from a wastewater sample after disinfection collected from Plant B in Norway (sidestream material processing plant). The isolate was identified as *Bacillus licheniformis*.

Resistance across isolates (*n* = 77)

Isolate group	AMP	CTX	TAZ	MEM	TMS	CIP	GEN	TET	CST	MDR*	
Escherichia coli - Plant D	8	4	0	0	4	10	3	nd	0	9	*n* = 10
Escherichia coli - Plant C	0	0	0	0	0	11	0	nd	0	0	*n* = 12
Other Enterobacterales	11	2	2	0	3	7	1	nd	0	5	*n* = 14
Aeromonas spp.	nd	nd	1	nd	2	2	nd	nd	nd	1	*n* = 3
Kurthia spp.	nd	nd	nd	nd	nd	nd	nd	nd	nd	0	*n* = 6
Acinetobacter spp.	nd	nd	nd	0	nd	0	2	nd	7	0	*n* = 15
Pseudomonas spp.	nd	nd	0	1	nd	0	nd	nd	0	0	*n* = 17

Number of isolates MDR* = Multidrug resistant

Supplementary Figure S3 Number of isolates (*n* = 77) with resistance against nine different antibiotics from seven classes. The selection includes Enterobacterales, *Aeromonas* spp., *Kurthia* spp., *Acinetobacter* spp. and *Pseudomonas* spp. Resistance was defined based on EUCAST clinical breakpoints, nd indicates that no data for breakpoints were available.

Functional Group	Key Genes							
Type III secretion & effectors	*eae-*	tir	espA	espB	nleA	nleB	cif	
Toxins	*astA*	hlyF	hlyE	tsh				
Adhesins & fimbriae	*fimH*	papC	tia	fdeC	etsC	iha	hra	
Iron uptake systems	*chuA*	fyuA	irp2	iroN	ireA	iutA	iucC	sitA
Capsules	*kpsE*	kpsMII_K4	kpsMII_K5					

O83:H42	O26:H34	O78:H17	O153:H19	O9:H11	O153:H19	O131:H12	O153:H19	O153:H19	O83:H42
CQW2.2	CQS1.1	CQB10.1	DES5.1	DQS6.1	DQW5.1	DQS3.1	DES10.1	DES7.1	DQS8.1
AslA	AslA	anr	anr	anr	anr	anr	anr	anr	AslA
air	anr	astA	cia	cib	cia	cea	cia	cia	air
anr	astA	cba	cib	cma	csgA	cib	cib	cib	anr
astA	cia	cea	csgA	csgA	etsC	csgA	csgA	csgA	astA
cea	cif	cia	etsC	cvaC	fdeC	cvaC	etsC	etsC	cea
chuA	csgA	cma	fdeC	fdeC	fimH	etsC	fdeC	fdeC	chuA
cib	cvaC	colE9	fimH	fimH	gad	fdeC	fimH	fimH	csgA
csgA	eae-	csgA	gad	gad	hlyE	fimH	gad	gad	cvaC
cvaC	e01-	fdeC	hlyE	hlyE	hlyF	gad	hlyE	hlyE	eilA
eilA	epsilon	fimH	hlyF	hlyF	hra	hha	hlyF	hlyF	espY2
espY2	espA	fyuA	hra	hra	iroN	hlyE	hra	hra	fdeC
etsC	espB	gad	iroN	iucC	iss	hlyF	iroN	iroN	fimH
fdeC	etsC	hha	iss	iutA	iucC	hra	iss	iss	hlyE
fimH	fdeC	hlyE	iucC	lpfA	iutA	iroN	iucC	iucC	hra
hha	fimH	hlyF	iutA	nlpI	lpfA	iss	iutA	iutA	iha
hlyE	gad	iha	lpfA	ompT	nlpI	iucC	lpfA	lpfA	iroN
hlyF	hha	irp2	nlpI	papC	ompT	iutA	nlpI	nlpI	iss
hra	hlyE	iucC	ompT	sitA	papC	lpfA	ompT	ompT	iucC
iha	hlyF	iutA	papC	terC	sitA	mchF	papC	papC	iutA
ireA	iroN	lpfA	sitA	tia	terC	neuC	sitA	sitA	kpsE
iroN	iss	nlpI	terC	traJ	traJ	nlpI	terC	terC	kpsMII_K4
iss	iucC	ompT	traJ	traT	traT	ompT	traJ	traJ	lpfA
iucC	iutA	papC	traT	yehA	tsh	shiA	traT	traT	mchF
iutA	mchF	shiA	tsh	yehB	yehA	shiB	tsh	tsh	mcmA
kpsE	nleA	sitA	yehA	yehC	yehB	sitA	yehA	yehA	nlpI
kpsMII_K5	nleB	terC	yehB	yehD	yehC	terC	yehB	yehB	ompT
lpfA	nlpI	tia	yehC		yehD	traJ	yehC	yehC	shiA
mchB	ompT	traT	yehD			traT	yehD	yehD	sitA
mchC	sitA	yehA				tsh			terC
mchF	terC	yehB				yehA			tia
mcmA	tia	yehC				yehB			traT
nlpI	tir	yehD				yehC			tsh
ompT	traJ					yehD			yehB
shiA	traT								yehC
shiB	tsh								yehD
sitA	yehA								yfcV
terC	yehB								
tia	yehC								
traT	yehD								
yehB									
yehC									
yehD									
yfcV									

Supplementary Figure S4 Predicted virulence genes in WGS-sequences of ten *Escherichia coli* isolates from broiler processing plants (Plant C and D). Results were obtained from the CGE (Center for Genomic Epidemiology) VirulenceFinder.

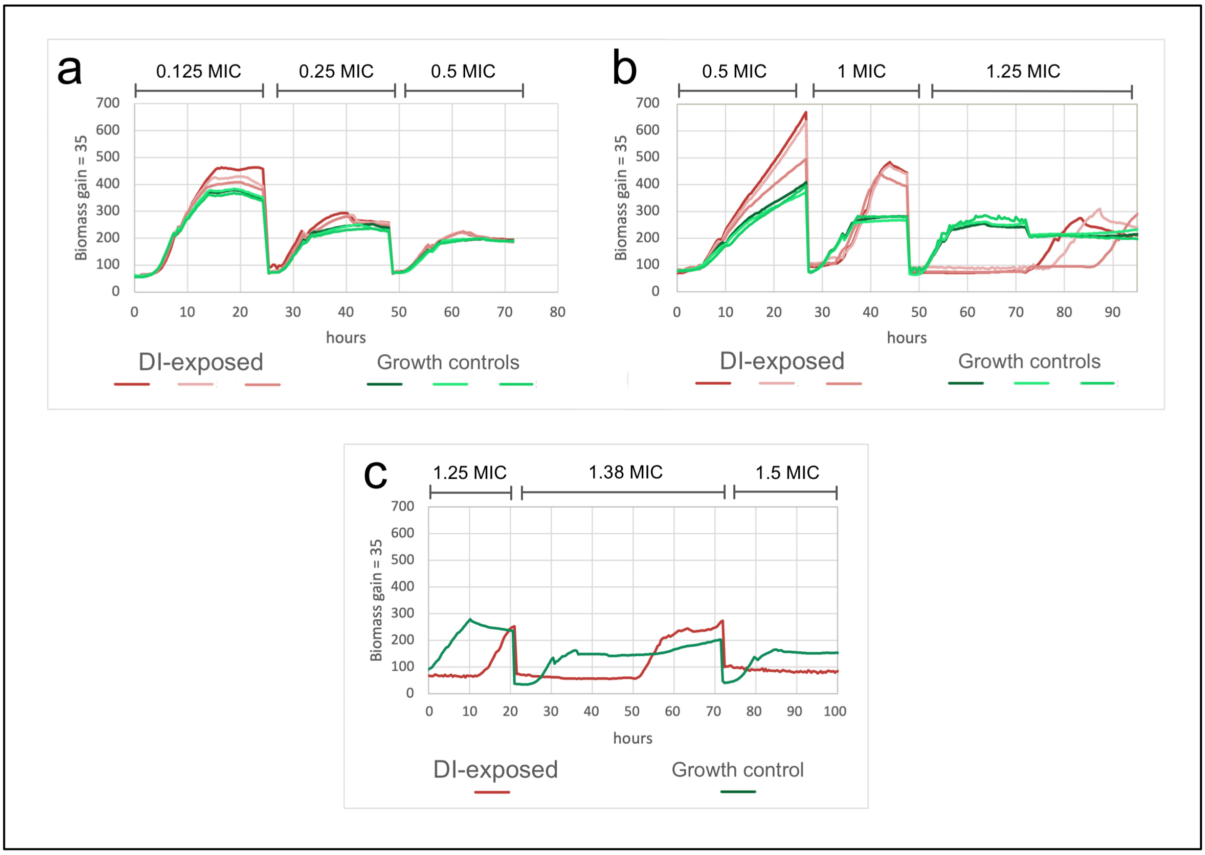

Supplementary Figure S5 Growth curves of isolate *Pseudomonas koreensis* 7A-4-2 at increasing concentrations of Aqua Des Foam PAA (ADF) from sub-MIC concentrations and above MIC. The growth controls were cultivated in MHB without DI. The DI-exposed cultures were incubated twice at some of the concentrations with a new batch of ADF to allow adaptation. a – first three incubation cycles from 0.125 – 0.5 MIC ADF. b – next three incubation cycles from 0.5 – 1.25 MIC ADF. c – one of the three replicates tolerated up to 1.38 MIC until complete inhibition at 1.5 MIC. Tests were performed in 96-well plates. The isolate 7A-4-2 was sampled in a drain by the fileting machine in a salmon processing plant after disinfection (Plant A).

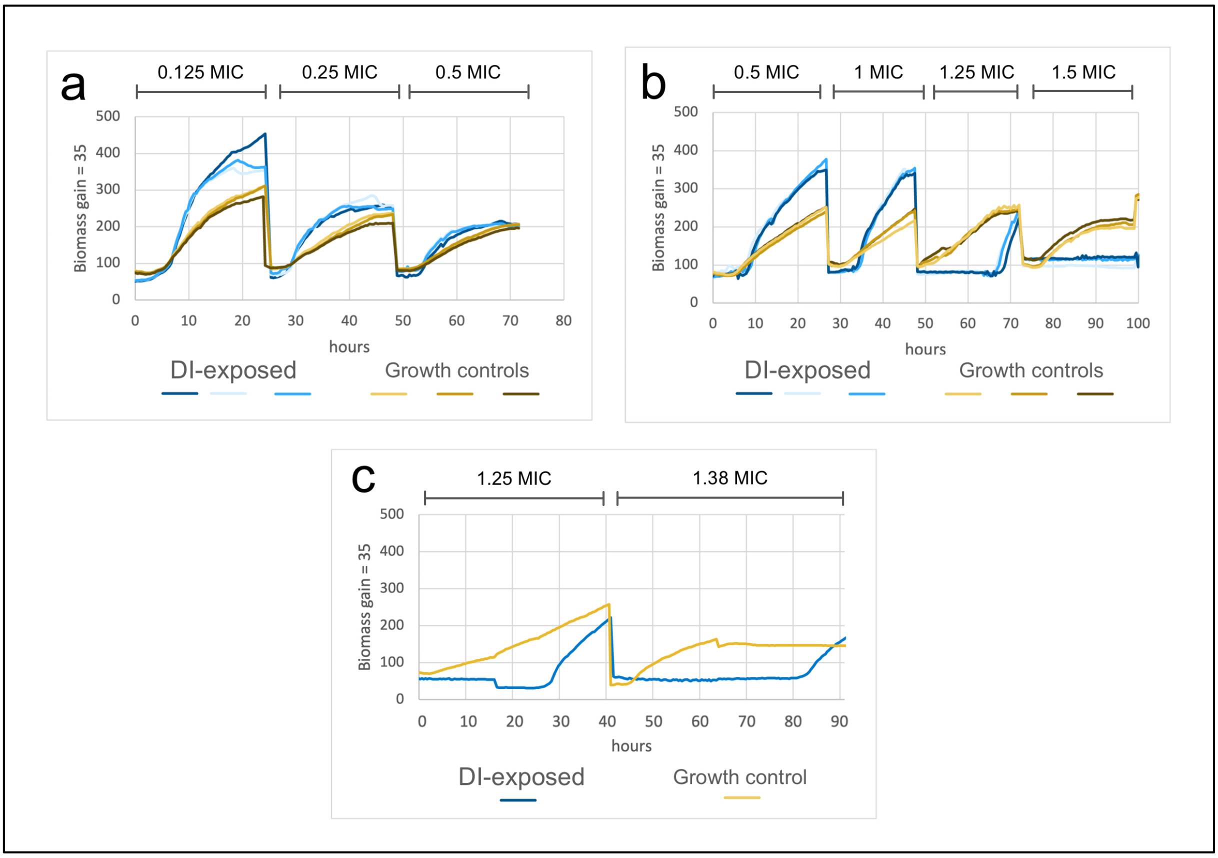

Supplementary Figure S6 Growth curves of isolate *Pseudomonas fragi* 10B-1-1 at increasing concentrations of Aqua Des Foam PAA (ADF) from sub-MIC concentrations and above MIC. The growth controls were cultivated in MHB without DI. The DI-exposed cultures were incubated twice at some of concentrations with a new batch of ADF to allow adaptation. a – first three incubation cycles from 0.125 – 0.5 MIC ADF. b – next three incubation cycles from 0.5 – 1.5 MIC ADF. c – one of the three replicates tolerated up to 1.38 MIC until complete inhibition at 1.5 MIC. Tests were performed in 96-well plates. The isolate 10B-1-1 was sampled inside a brining vessel in a broiler processing plant before disinfection (Plant C).

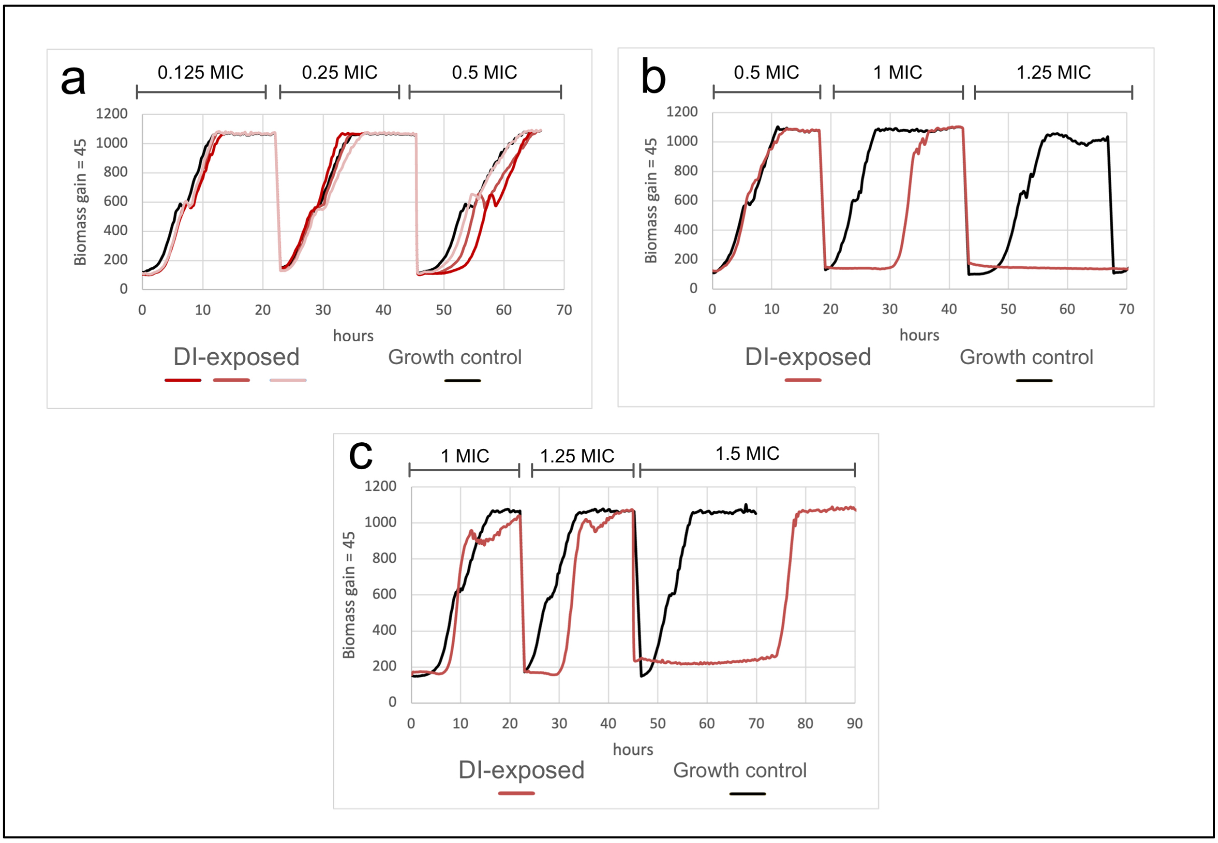

Supplementary Figure S7 Growth curves of isolate *Pseudomonas koreensis* CFC10A-1-1 at increasing concentrations of Aqua Des Foam PAA (ADF) from sub-MIC concentrations and above MIC. The growth controls were cultivated in MHB without DI. The DI-exposed cultures were incubated twice at some of concentrations with a new batch of ADF to allow adaptation. a – first three incubation cycles from 0.125 – 0.5 MIC ADF. b – one of the three replicates next three incubation cycles from 0.5 – 1.25 MIC ADF. c – one of the three replicates tolerated up to 1.5 MIC until complete inhibition at 1.75 MIC. Tests were performed in 48-well plates. The isolate CFC10A-1-1 was sampled from a crate washer in a salmon processing plant after disinfection (Plant A).
